# Supplementary material for: A cocoon silk chemistry strategy to ultrathin N-doped carbon nanosheet with metal single-site catalysts
Source: Nat Commun. 2018 Sep 21;9:3861. doi: 10.1038/s41467-018-06296-w (PMC6155020; doi:10.1038/s41467-018-06296-w)
Supplement: Supplementary file 1 — Supplementary Information [file 41467_2018_6296_MOESM1_ESM.pdf]

Supplementary Information

A cocoon silk chemistry strategy to ultrathin N-doped carbon nanosheet with metal single-site catalysts

Zhu et al.

Materials and Methods

Reagents and Materials

All chemical reagents were used as received without further purification. *Bombyx mori* cocoons were purchased from Huangshan Huasheng Silk Group Co., Ltd. Cobalt chloride hexahydrate ($CoCl_2·6H_2O$), ferric chloride hexahydrate ($FeCl_3·6H_2O$), nickel chloride hexahydrate ($NiCl_2·6H_2O$), sodium carbonate (Na_2CO_3), zinc chloride ($ZnCl_2$), hydrochloric acid (HCl), cobalt acetate, melamine, and sodium chloride (NaCl) were purchased from Sinopharm Chemical Reagent Co., Ltd. H_2O_2 (30 wt%) was purchased from Beijing Chemical Works. Benzene was purchased from Alfa Aesar (China) Chemicals Co., Ltd. MeCN (extra pure), cobalt phthalocyanine, and n-hexadecane were purchased from Acros Organics.

Synthesis of melamine-derived Co-N-C material

To investigate the role of carbon precursor in shaping the morphology of final pyrolysis product, nitrogen-containing melamine was employed to replace the regenerated silk fibroin. Briefly, 6 g of melamine was first dispersed in 100 mL aqueous solution of 0.25 M $CoCl_2$ and 0.11 M $ZnCl_2$ under continuously stirring. The subsequent treatment procedures were the same as that for preparing Co-ISA/CNS.

Synthesis of pure N-doped carbon

The pure N-doped carbon was synthesized by the same procedures as that for preparing Co-ISA/CNS, except using 100 mL NaCl (0.25 M) aqueous solution to replace 100 mL aqueous solution of 0.25 M $CoCl_2$ and 0.11 M $ZnCl_2$. The subsequent treatment procedures were the same as that for preparing Co-ISA/CNS.

Synthesis of Co-NPs

For comparison, the Co nanoparticles supported on N-doped carbon were also synthesized by carbon thermal reduction. 0.06 g of cobalt acetate and 0.2 g of the above-mentioned N-doped carbon were ground thoroughly in an agate mortar for 1 hour. The obtained mixtures were transferred into a corundum boat and then heated to 900 °C with heating rate of 3°C min^{-1} for 1 h in Ar-saturated tube furnace. After that the system was naturally cooled down to room temperature. The obtained products were washed by distilled water and anhydrous ethanol for several times. The final sample was collected by centrifugation and then dried in oven overnight.

Materials Characterization

The crystallographic phase of the as-synthesized materials was characterized by X-ray powder diffractometer (XRD, Rigaku RU-200b, CuKα source, λ = 1.5418 Å). Themicrostructures and morphology of the as-synthesized materials were identified by field emission scanning electron microscopy (FESEM, Hitachi SU8010), and transmission electron microscopy (TEM, Hitachi H-800, 100 kV), and high-resolution TEM (HRTEM, JEOL 2100F, 200 kV) equipped with energy-dispersive X-ray spectroscopy (EDS, Oxford Instruments, INCA). The STEM characterizations were performed on a transmission electron microscope with a probe corrector (AC-STEM, FEI, Titan Cubed Themis G2 300, 300 kV). The specific surface areas and pore size distribution were evaluated by nitrogen adsorption-desorption isotherms on a NOVA4200e nitrogen adsorption apparatus (Quantachrome Instruments, USA) at 77 K under a relative pressure p/p_0 of 3.3×10^{-7} – 0.995. Prior to the measurement, the samples were degassed at 574 K for 6 h under vacuum to remove adsorbed gas or moisture. The surface area was estimated by the multi-point Brunauer−Emmett−Teller (BET) theory. Pore size distribution was calculated from the adsorption data using Barrett–Joyner–Halenda (BJH) model. X-ray photoelectron spectra (XPS) were recorded on a ULVAC PHI Quantera microscope machine. The standard binding energies (284.6 eV) of C1s peak was selected to calibrate experimental date. Raman spectra were taken using a Renishaw inViaTM confocal Raman microscope (Renishaw, England) with a 514 nm excitation laser under an intensity of 5 mW. The inductively coupled plasma atomic emission (ICP-AES) was conducted on Thermo Fisher IRIS Intrepid Ⅱ.The metal species was extracted through a combustion method. Electron paramagnetic resonance EPR spectra were taken at 123 K using a JES-FA200 ESR Spectrometer. The required experimental parameters were set as follows: microwave Frequency = 9035.960 MHz, microwave power = 1.0 mW, modulation amplitude = 1000 mT, modulation frequency = 100.00 kHz, and time constant = 0.3 s.

X-ray absorption fine structure spectroscopy measurements

The X-ray absorption fine structure spectra were performed at 1W1B station in Beijing Synchrotron Radiation Facility (BSRF, operated at 2.5 GeV with a maximum current of 250 mA). The XAFS data were recorded in fluorescence excitation mode using a Lytle detector, and the spectra of all references were collected in the transmission mode. All samples were pelletized as

disks of 13 mm diameter with about 1 mm thickness using polyvinylidene fluoride as binder. The acquired XAFS data were analyzed by Athena and Artemis software, according to the standard procedures. The k^3-weighted EXAFS spectra were obtained by subtracting the post-edge background from the overall absorption and then normalizing with respect to the edge-jump step. Subsequently, χ (k) data of Co K-edge in the k-space from 2.7-11.1 Å^{-1} were Fourier transformed to real (R) space using a hanning windows (dk=1.0 Å^{-1}) to separate the EXAFS contributions from different coordination shells. To obtain the quantitative structural parameters around Co atoms, least-squares curve parameter fitting was performed. Effective scattering amplitudes and phase-shifts for the Co–N and Co–Co pairs were calculated with the ab initio code FEFF9. The amplitude reduction factor S_0^2 was obtained by fitting Co foil and Co oxide counterparts. In the subsequent fitting of Co-ISA/CNS, the coordination numbers N, interatomic distances R, Debye-Waller factor σ^2 and the edge-energy shift ΔE_0 were allowed to run freely.

Computation methods

The calculations in this study were performed using the plane-wave pseudopotential method within the framework of DFT. The ion core and valence electron interaction was described by using a Vanderbilt-type ultrasoft pseudopotential[1]. The exchange-correlation interactions were treated by using the generalized-gradient approximation (PBE/GGA) scheme[2]. The plane wave cutoff energy was set to 400 eV. The Co-ISA/CNS model was set in a 4 × 4 supercell of graphene with a vacuum thickness of 15 Å between graphene layers. The Brillouin zone integrations were performed over 2 × 2 × 1 grid points using the Monkhorst-Pack scheme for Co-ISA/CNS based systems. The Co-NPs and CoPc models were set in a 15 × 15 × 15 Å cubic box, and their Brillouin zone integrations were performed over 1 × 1 × 1 grid point. The Co-NP was modeled by an isolated octahedral Co_6 cluster. Spin polarization was considered in all calculations. All atoms were relaxed to their equilibrium positions when the change in energy on each atom between successive steps was converged to 1×10^{-5} eV. The forces on each atom were converged to 0.03 eV Å^{-1}. The stress on each atom was converged to 0.05 GPa and the displacement was converged to 0.001 Å. The transition states were searched by complete Linear Synchronous Transit (LST) and Quadratic Synchronous Transit (QST) methods. All the total energy calculations were performed in reciprocal space by using the CASTEP code[3].

Supplementary Figures and Tables

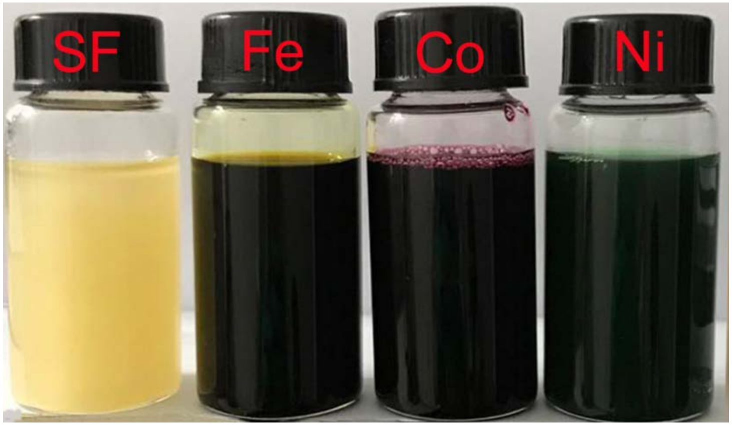

Supplementary Figure 1 | Photographs of the as-obtained typical regenerated silk fibroin solutions prepared by dissolving degummed natural silk using concentrated salt solution of NaCl, $FeCl_3 \cdot 6H_2O/ZnCl_2$, $CoCl_2 \cdot 6H_2O/ZnCl_2$, and $NiCl_2 \cdot 6H_2O/ZnCl_2$, respectively.

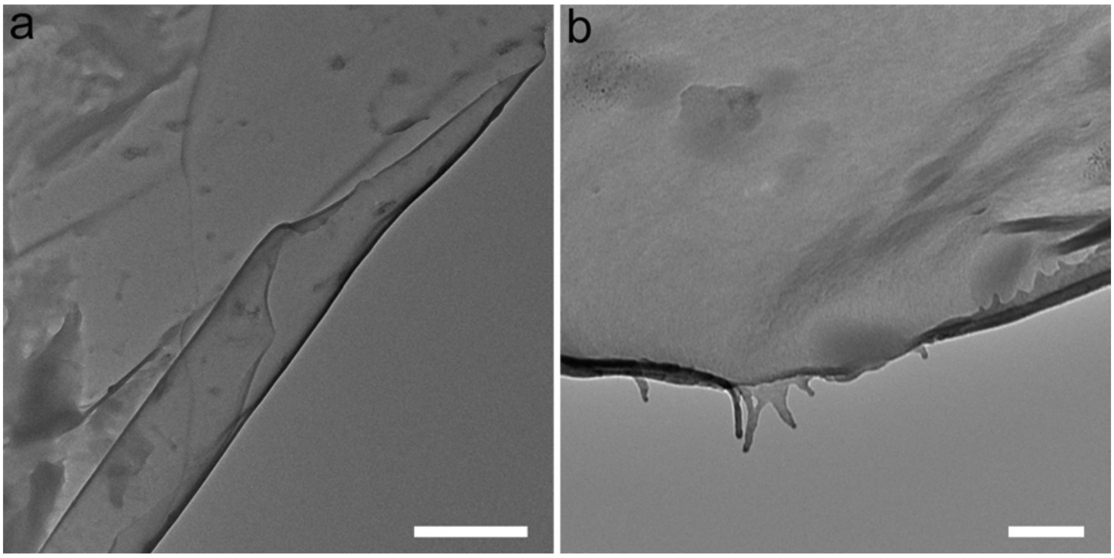

Supplementary Figure 2 | Typical low-magnification (**a**) and magnified (**b**) TEM images of regenerated silk fibroin precursor. Scale bar, 1 μ and 200 nm.

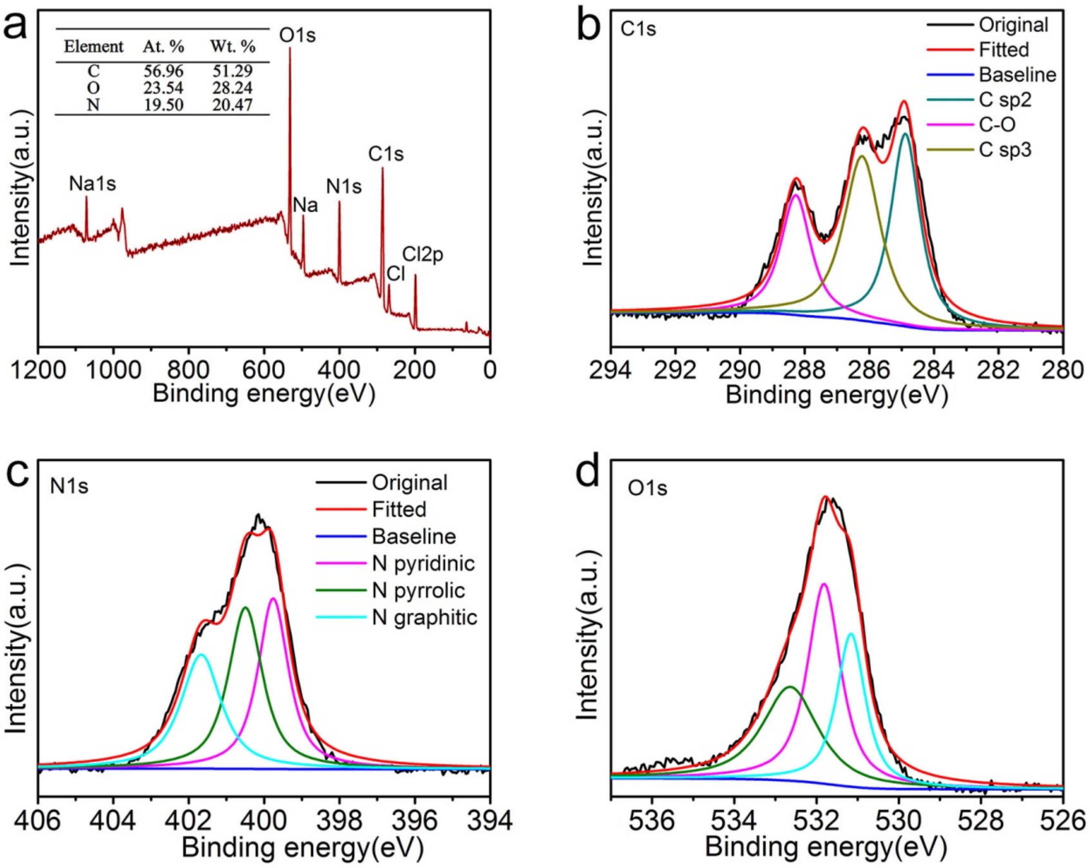

Supplementary Figure 3 | XPS spectra of regenerated silk fibroin precursor dissolved in NaCl (0.4 M). Survey spectrum (**a**), high-resolution (**b**) C 1s, (**c**) N 1s, and (**d**) O 1s spectra, the inset in (**a**) showing the chemical identification of regenerated silk fibroin precursor.

XPS spectra well demonstrate a rich chemistry of silk fibroin. The rich compositional element nitrogen from amino groups accounts for almost 20.47 wt %.

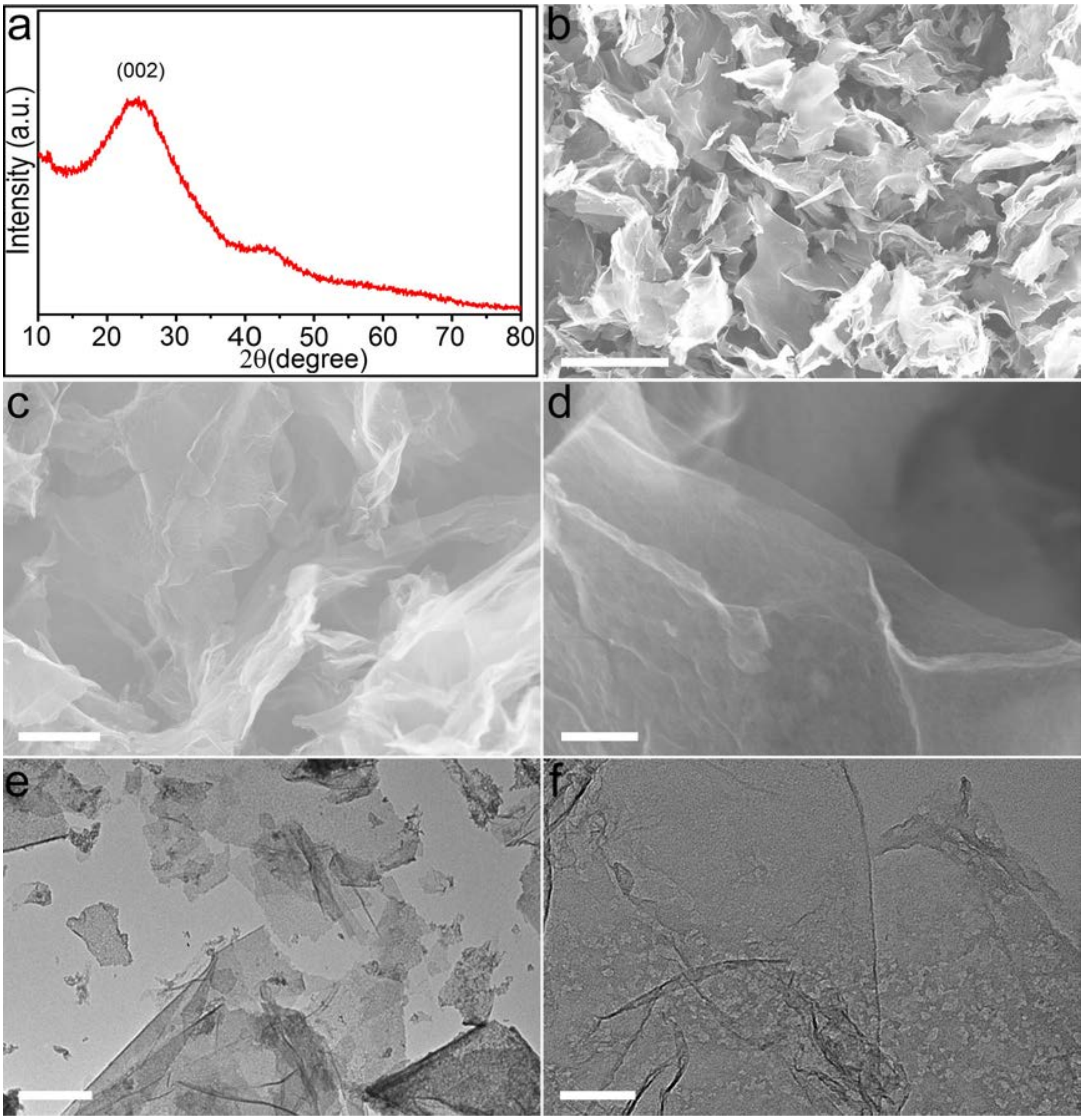

Supplementary Figure 4 | Microstructure and morphology characterizations of Co-ISA/CNS. (**a**) XRD pattern. (**b**) and (**c**) Typical low-magnification FESEM images. Scale bar, 5 and 1 μm. (**d**) Magnified FESEM image. Scale bar, 200 nm. (**e**) and (**f**) TEM images. Scale bar, 5 1 μm and 100 nm.

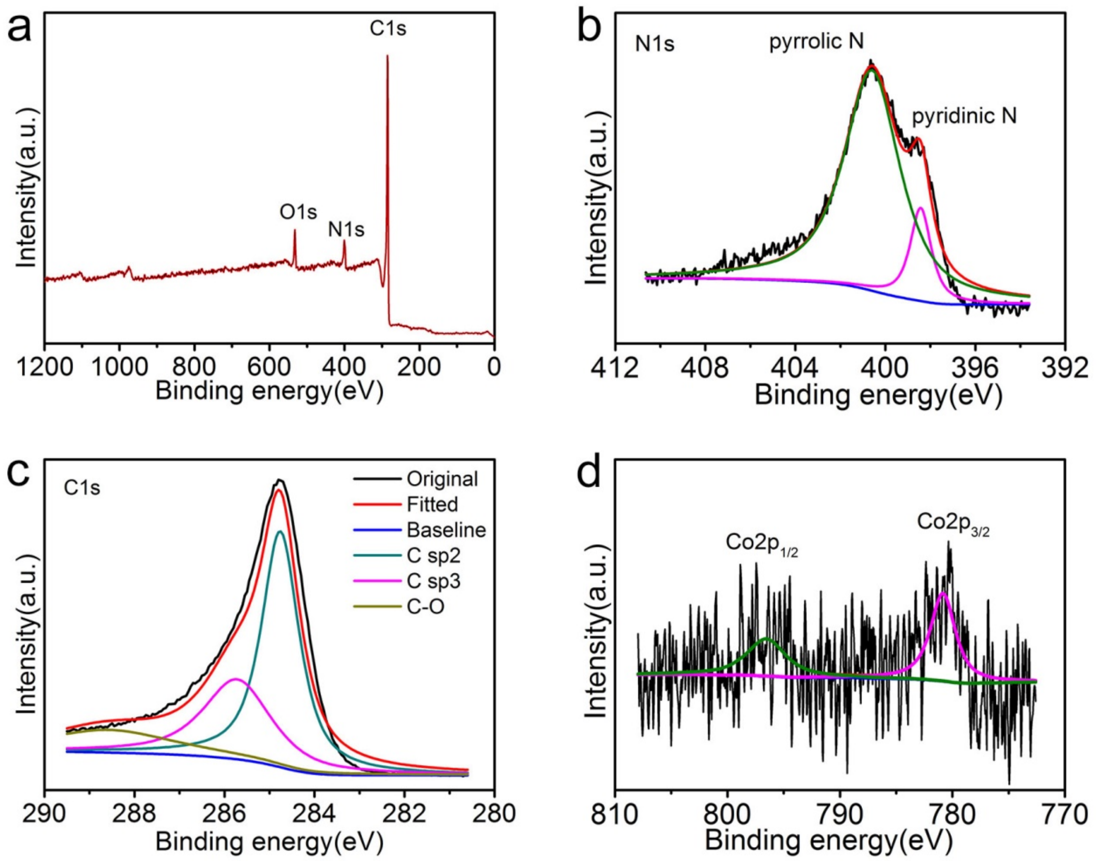

Supplementary Figure 5 | XPS spectra of Co-ISA/CNS. survey spectrum (**a**), high-resolution (**b**) N 1s, (**c**) C 1s, and (**d**) Co 2p spectra.

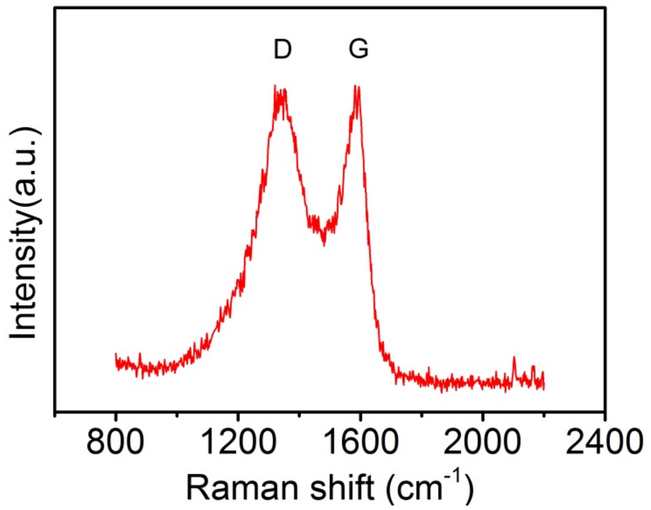

Supplementary Figure 6 | Raman spectrum of Co-ISA /CNS.

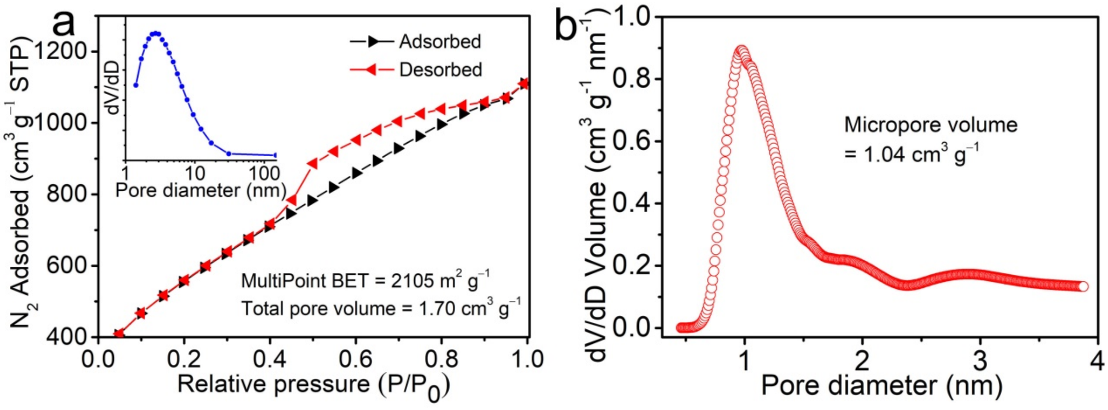

Supplementary Figure 7 | (**a**) Nitrogen adsorption-desorption isotherms and the corresponding pore size distribution (inset and **b**) of Co-ISA/CNS catalyst.

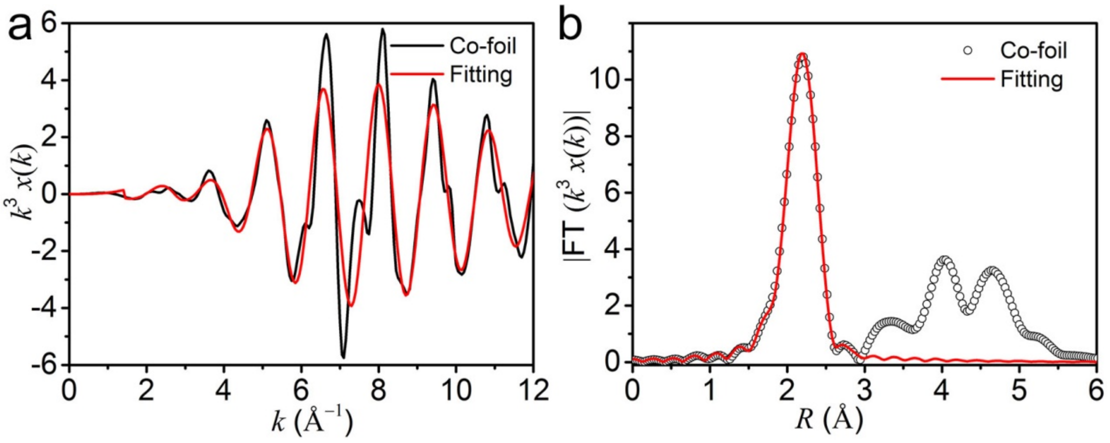

Supplementary Figure 8 | The corresponding EXAFS fitting curves of Co foil. (**a**) at *k* space and (**b**) at *R* space, respectively.

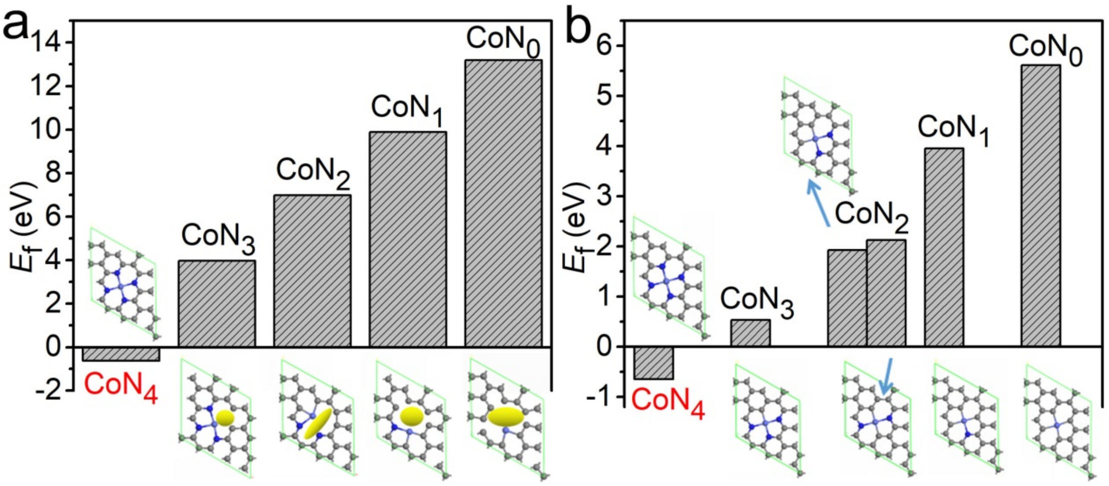

Supplementary Figure 9 | (**a**) and (**b**) Calculated structural stability of Co-ISA/CNS catalysts with various N coordination numbers in different CoN_x models (x = 0, 1, 2, 3, and 4).

Formation energy calculation

The formation energy is defined as followed:

$E_f = E_{tot} - n_C\mu_C - n_{Co}\mu_{Co} - n_N\mu_N$ (Supplementary Equation 1)

where E_{tot} is the total energy of the structure, n_C, n_{Co} and n_N are the number of carbon, cobalt, and nitrogen atoms in the studied structures, respectively, and $\mu_{C,}$ $\mu_{Co,}$ and μ_N are the chemical potentials of carbon, cobalt and nitrogen atoms relative to graphite, pure *hcp*-Co metal and nitrogen molecule. A negative value denotes the structural stability with respect to the elemental constituents. The constructed models in Supplementary Fig. 9a are consisted with those proposed in Deng's work[4]. The CoN_4 structure is constructed by removing two carbon atoms of graphene to form one vacancy firstly, then replacing the four nearest carbon atoms around vacancy by nitrogen atoms, finally putting metal atom in the vacancy site. As for other CoN_x (x = 1, 2, and 3), the targeted structures are constructed by removing the corresponding nitrogen atoms in CoN_4 model. The golden balls are used to highlight the holes in the structures. For the model in Supplementary Fig. 9b, the CoN_4 is constructed as the same as Deng's work. As for CoN_x (x = 1, 2, and 3), the structures are constructed by replacing the corresponding nitrogen atoms by carbon atoms in CoN_4 model to make a denser packing structure.

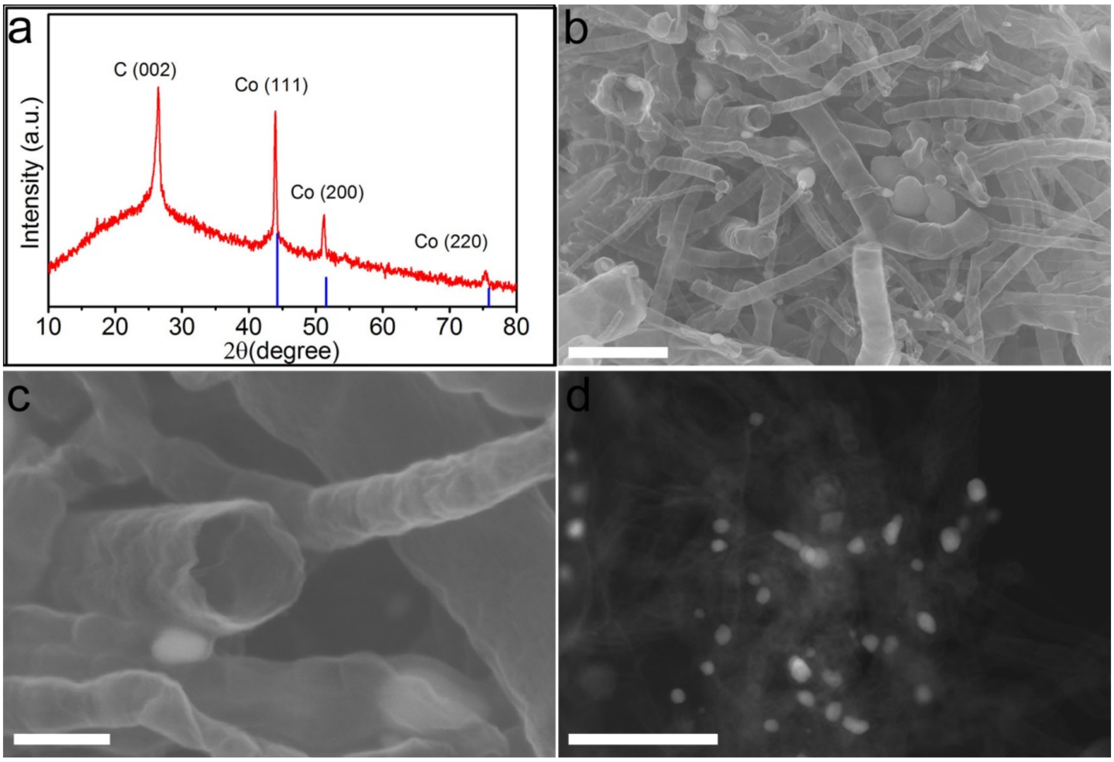

Supplementary Figure 10 | Microstructure and morphology characterizations of the pyrolytic sample converted from alternative melamine precursor. (**a**) XRD pattern with standard Co (JCPDS No. 15-0806, blue vertical line) as reference. (**b**) Typical low-magnification FESEM image. Scale bar, 500 nm. (**c**) Magnified FESEM image. Scale bar, 100 nm. (**d**) HAADF STEM image. Scale bar, 200 nm.

XRD pattern shows that the melamine derived sample consists of graphitic carbon and metallic cobalt. FESEM images HAADF STEM image reveal that the melamine derived sample displays a geometrical tube-like 1D microstructure with Co nanoparticles encapsulated at the tip of carbon nanotube. The results suggest that these two organic precursors undergo different pyrolysis behavior even though at the same heat treatment condition. The small molecule melamine may first degraded into disordered carbonaceous radicals, and then the resultant fragment were rearranged in 1D direction catalyzed by Co nanoparticles during pyrolysis. The Co nanoparticles were tightly encapsulated by multiple carbon layers and could survive in acid leaching process. For regenerated silk fibroin precursor, their lamella-like layer 2D backbone could be maintained in pyrolysis process. The reduced Co nanoparticles were anchored on surface of converted carbon layer and could be removed by acid leaching.

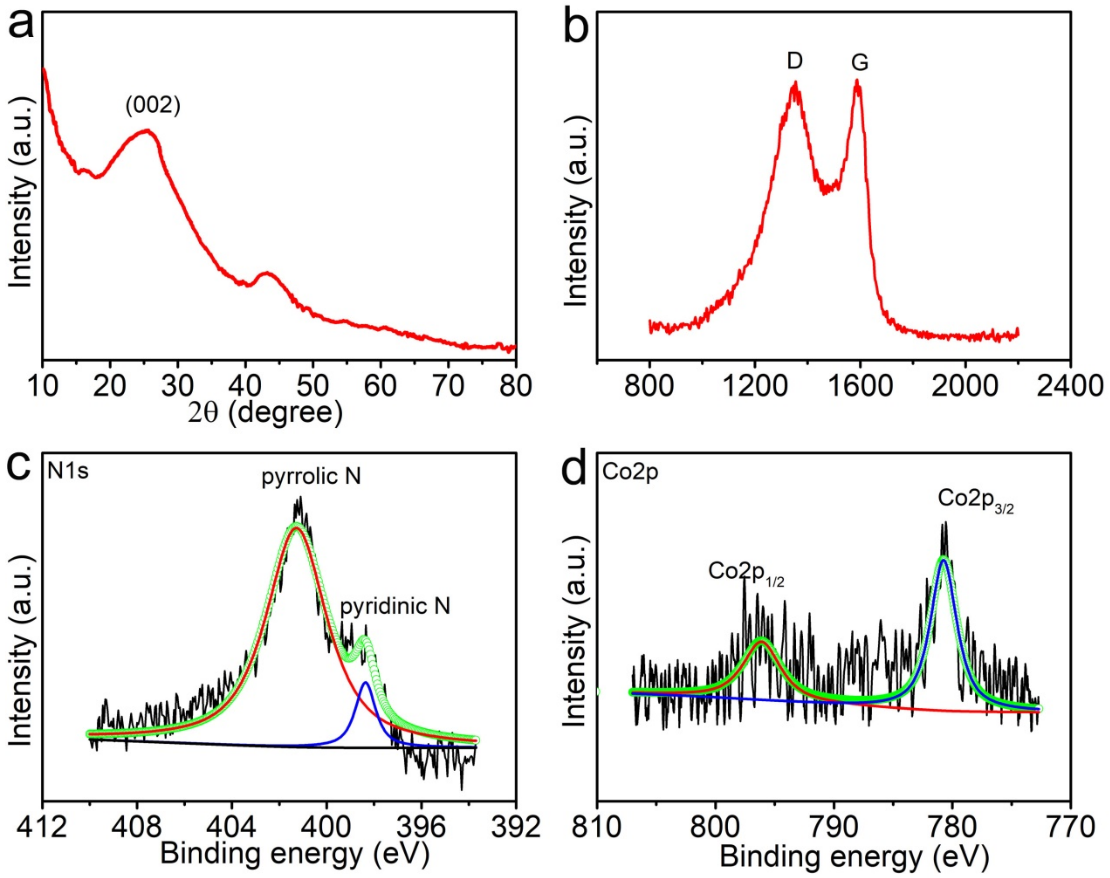

Supplementary Figure 11 | Chemical composition and physical characterizations of Co-ISA/CNS-3 catalyst. (**a**) XRD pattern. (**b**) Raman spectrum. (**c**) and (**d**) high-resolution N1s and Co 2p XPS spectra.

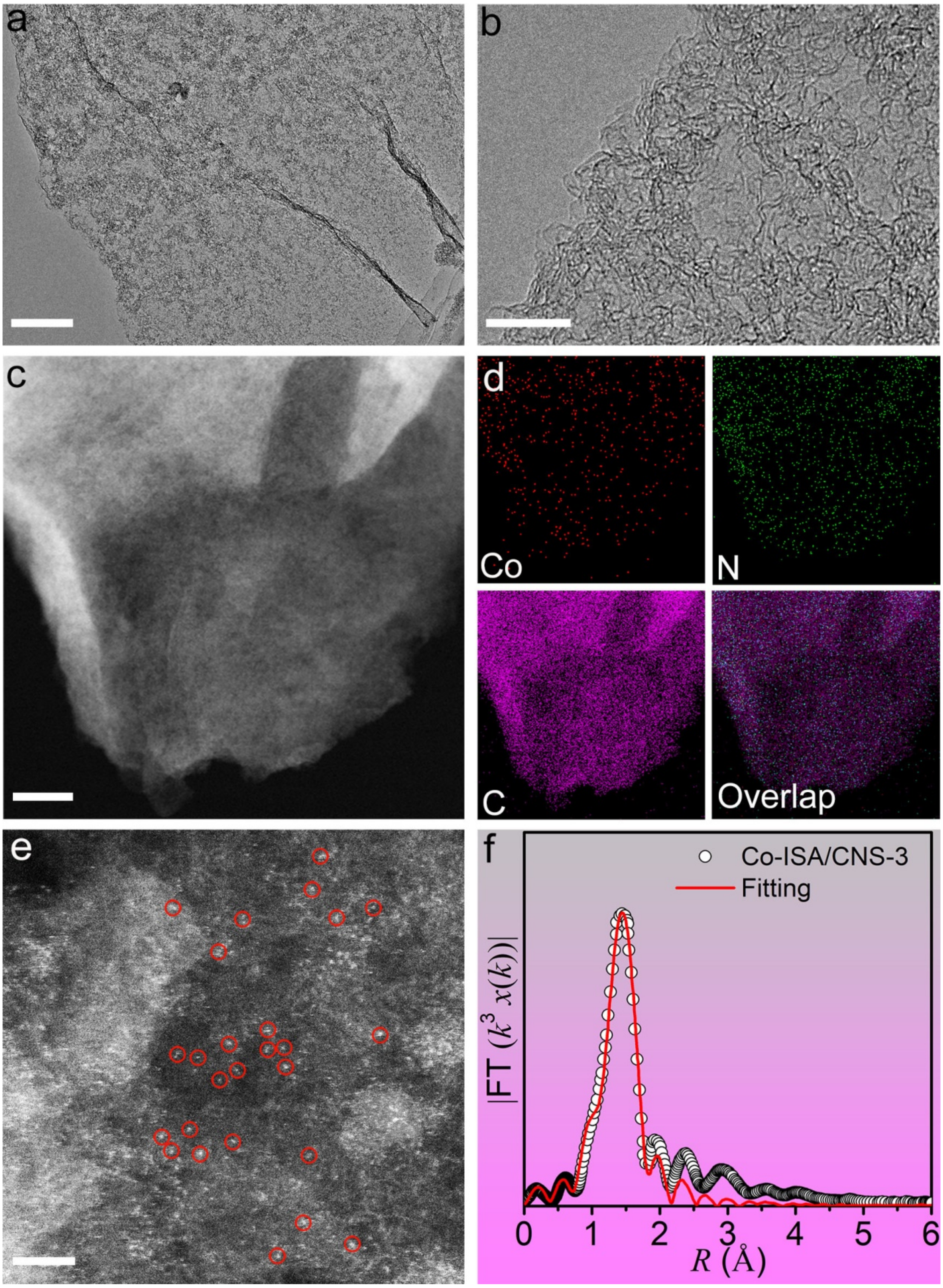

Supplementary Figure 12 | Microstructure and morphology characterizations of Co-ISA/CNS-3 catalyst. (**a**) TEM image. Scale bar, 500 nm. (**b**) HRTEM image. Scale bar, 10 nm. (**c**) and (**d**) HAADF STEM image and corresponding EDX elemental mapping. Scale bar, 500 nm. (**e**) Aberration-corrected HAADF-STEM image. Scale bar, 2 nm. (**f**) Fourier transformed (FT) k^3-

weighted χ(*k*)-function of the EXAFS spectra for Co K-edge and corresponding EXAFS fitting curves at *R* space.

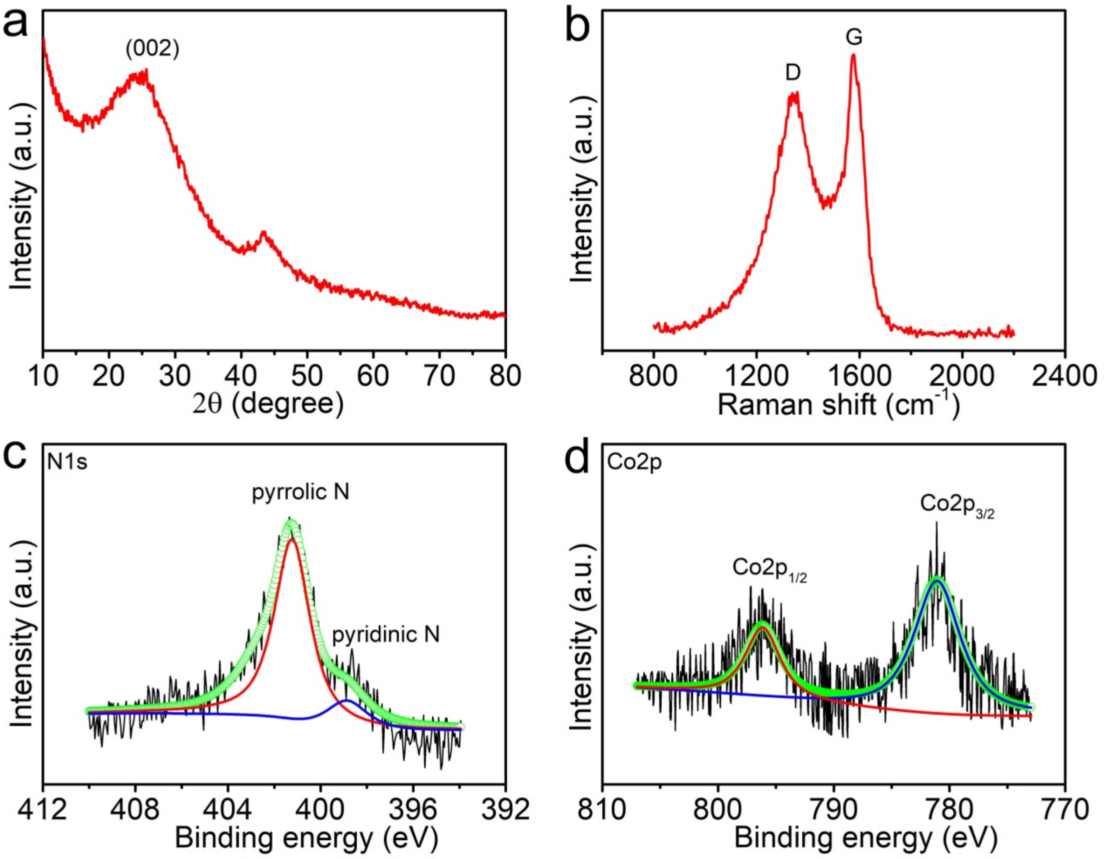

Supplementary Figure 13 | Chemical composition and physical characterizations of Co-ISA/CNS-2 catalyst. (**a**) XRD pattern. (**b**) Raman spectrum. (**c**) and (**d**) high-resolution N1s and Co 2p XPS spectra.

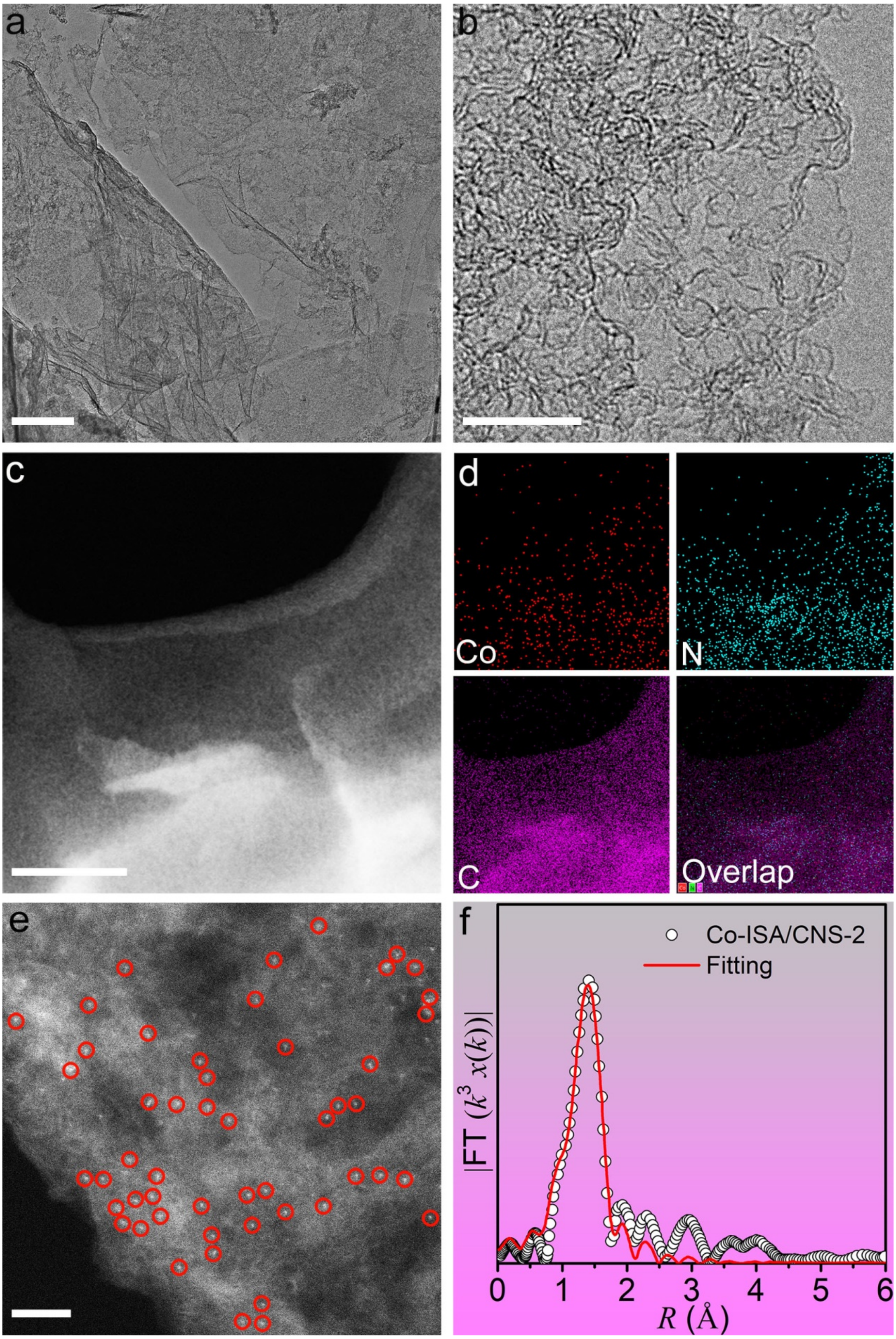

Supplementary Figure 14 | Microstructure and morphology characterizations of Co-ISA/CNS-2 catalyst. (**a**) TEM image. Scale bar, 500 nm. (**b**) HRTEM image. Scale bar, 10 nm. (**c**) and (**d**) HAADF STEM image and corresponding EDX elemental mapping. Scale bar, 500 nm. (**e**)

Aberration-corrected HAADF-STEM image. Scale bar, 2 nm. (**f**) Fourier transformed (FT) k^3-weighted $\chi(k)$-function of the EXAFS spectra for Co K-edge and corresponding EXAFS fitting curves at *R* space.

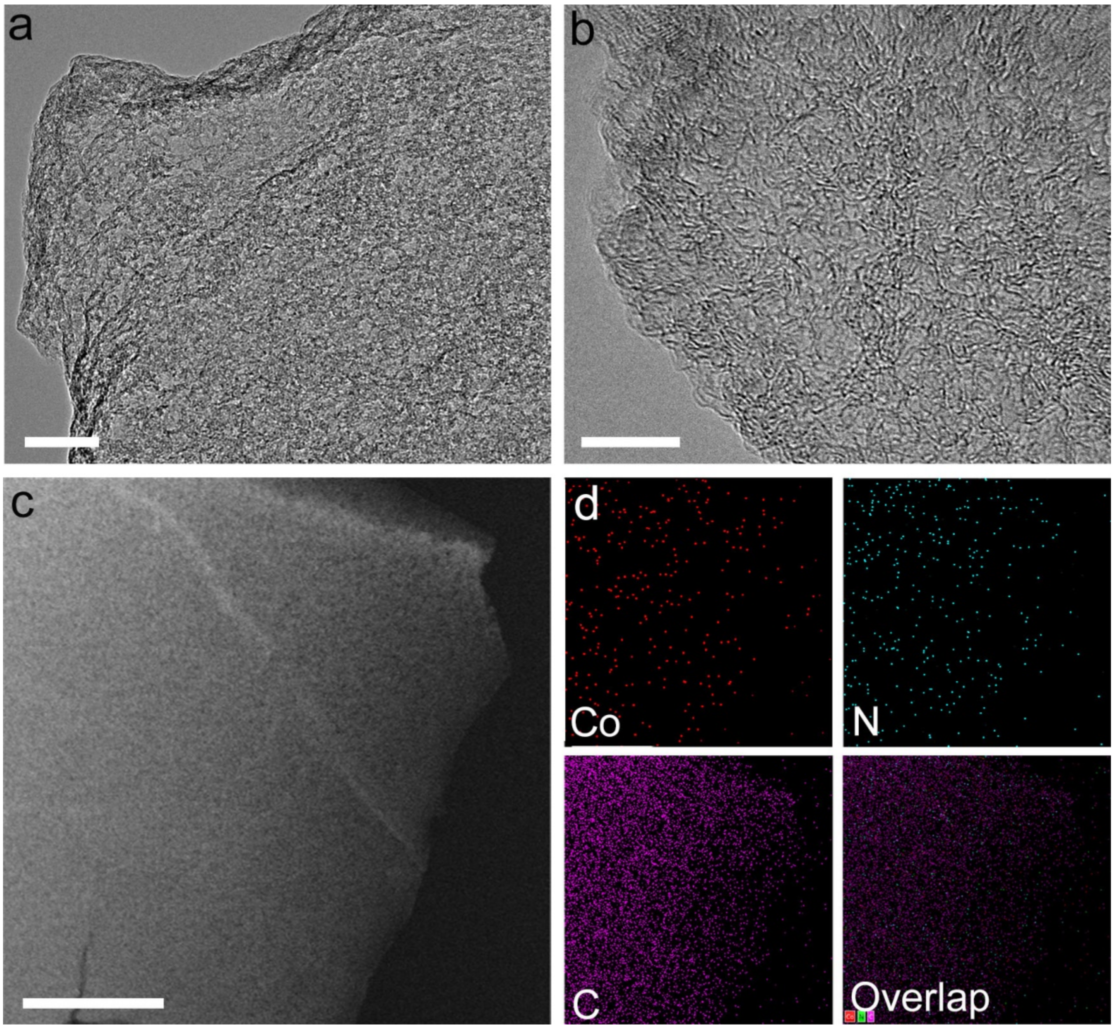

Supplementary Figure 15 | Microstructure and morphology characterizations of Co-ISA/CNS catalyst synthesized with 5.5 mmol $ZnCl_2$. (**a**) Typical TEM image. Scale bar, 100 nm. (**b**) HRTEM image. Scale bar, 10 nm. (**c**) HAADF-STEM image and (**d**) corresponding EDX elemental mapping. Scale bar, 500 nm.

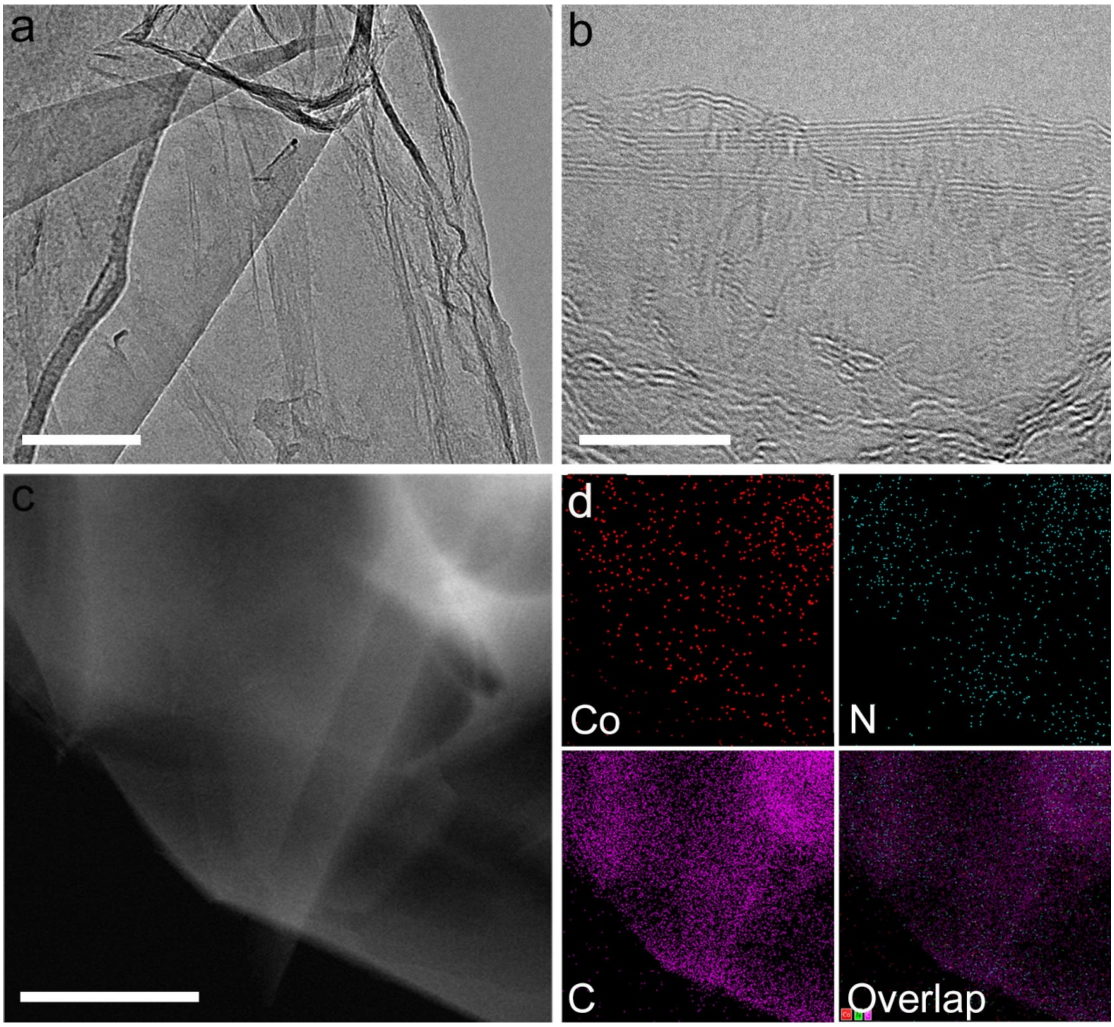

Supplementary Figure 16 | Microstructure and morphology characterizations of Co-ISA/CNS catalyst synthesized without $ZnCl_2$. (**a**) Typical TEM image. Scale bar, 200 nm. (**b**) HRTEM image. Scale bar, 10 nm. (**c**) HAADF-STEM image and (**d**) corresponding EDX elemental mapping. Scale bar, 500 nm.

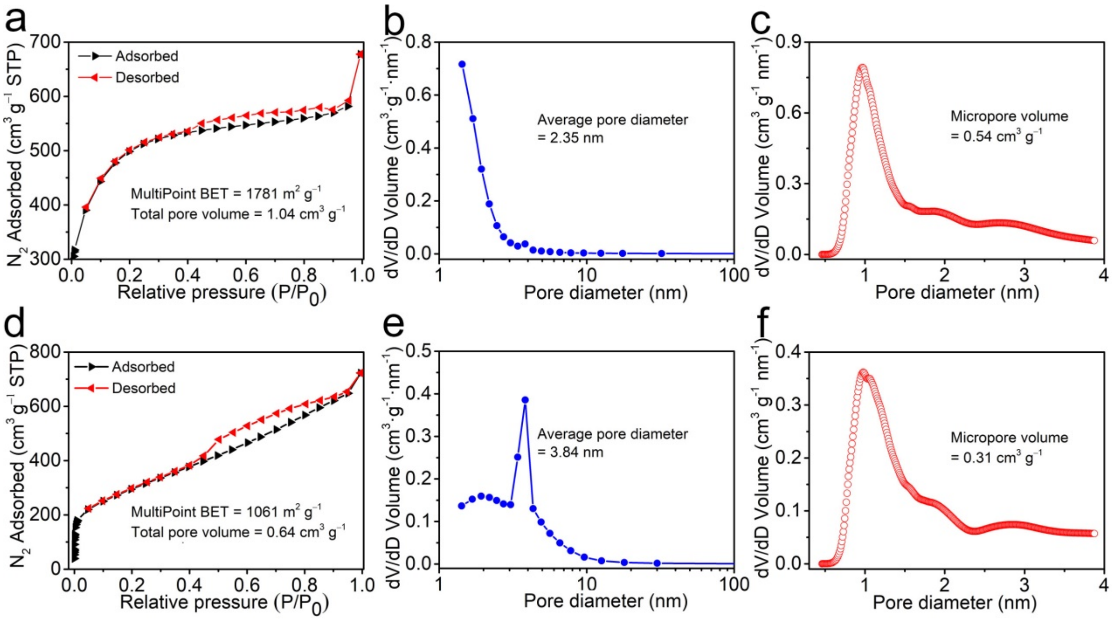

Supplementary Figure 17 | (**a**), (**b**), and (**c**) Nitrogen adsorption-desorption isotherms and the corresponding pore size distribution of Co-ISA/CNS catalyst synthesized with 5.5 mmol $ZnCl_2$, (**d**), (**e**), and (**f**) Nitrogen adsorption-desorption isotherms and the corresponding pore size distribution of Co-ISA/CNS catalyst synthesized without $ZnCl_2$.

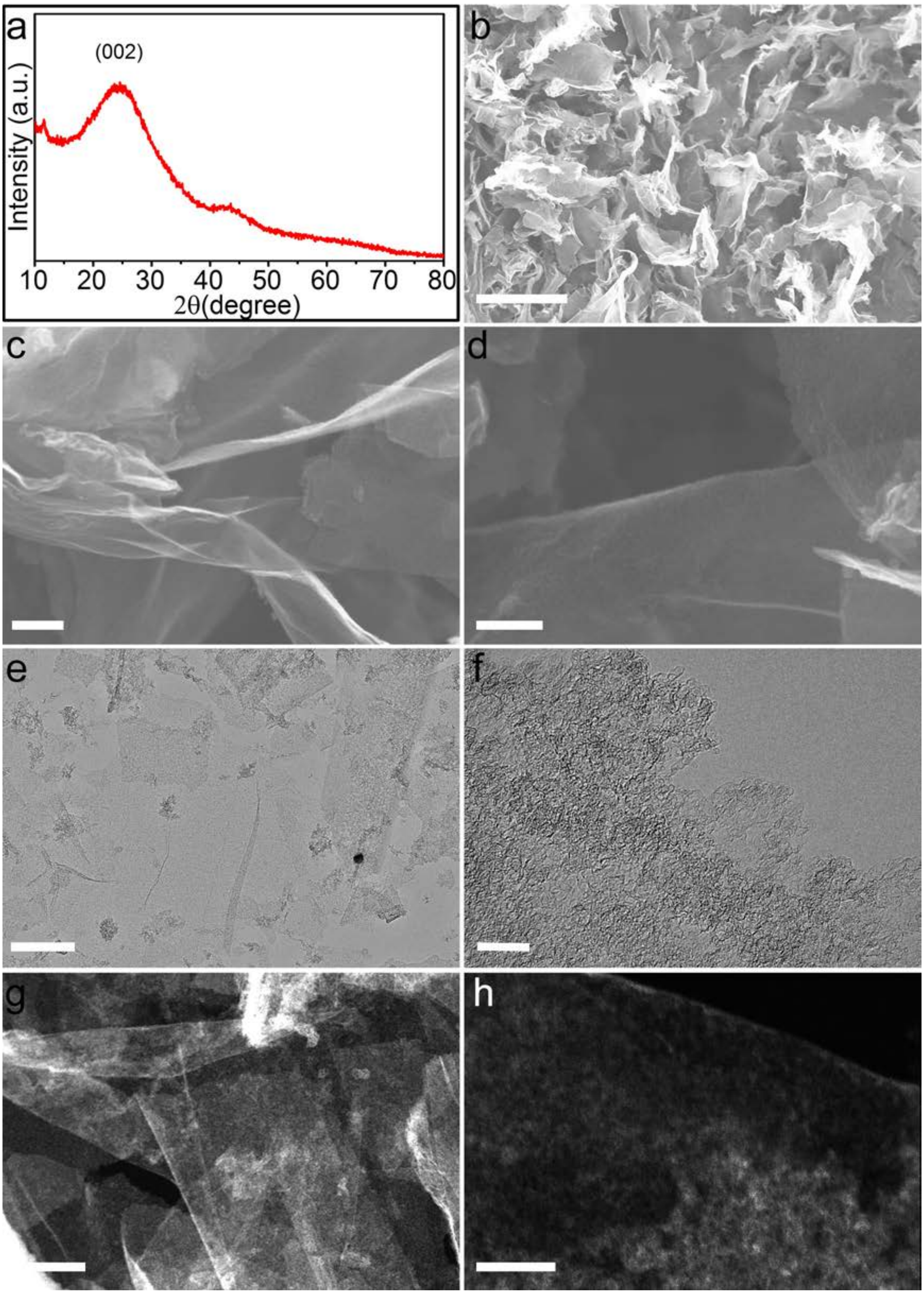

Supplementary Figure 18 | Microstructure and morphology characterizations of Fe-ISA/CNS. (**a**) XRD pattern. (**b**), (**c**), and (**d**) Typical FESEM images at different magnifications. Scale bar, 5 μ , 200 and 200 nm. (**e**) and (**f**) TEM and HRTEM images. Scale bar, 500 and 10 nm. (**g**) and (**h**) HAADF STEM images. Scale bar, 500 and 100 nm.

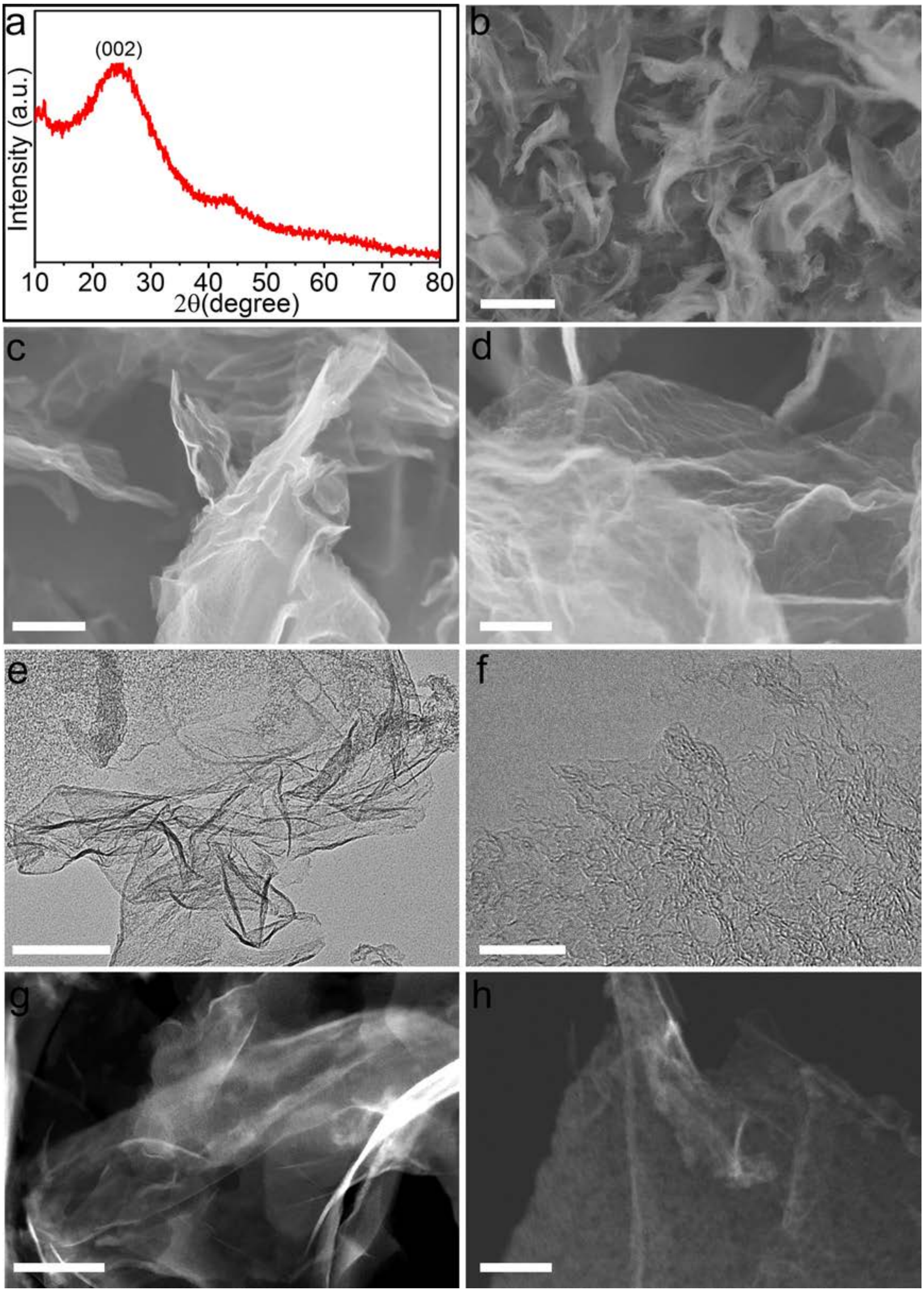

Supplementary Figure 19 | Microstructure and morphology characterizations of Ni-ISA/CNS. (**a**) XRD pattern. (**b**), (**c**), and (**d**) typical FESEM images at different magnifications. Scale bar, 2 μm, 500 and 200 nm. (**e**) and (**f**) TEM and HRTEM images. Scale bar, 200 and 10 nm. (**g**) and (**h**) HAADF STEM images. Scale bar, 500 and 100 nm.

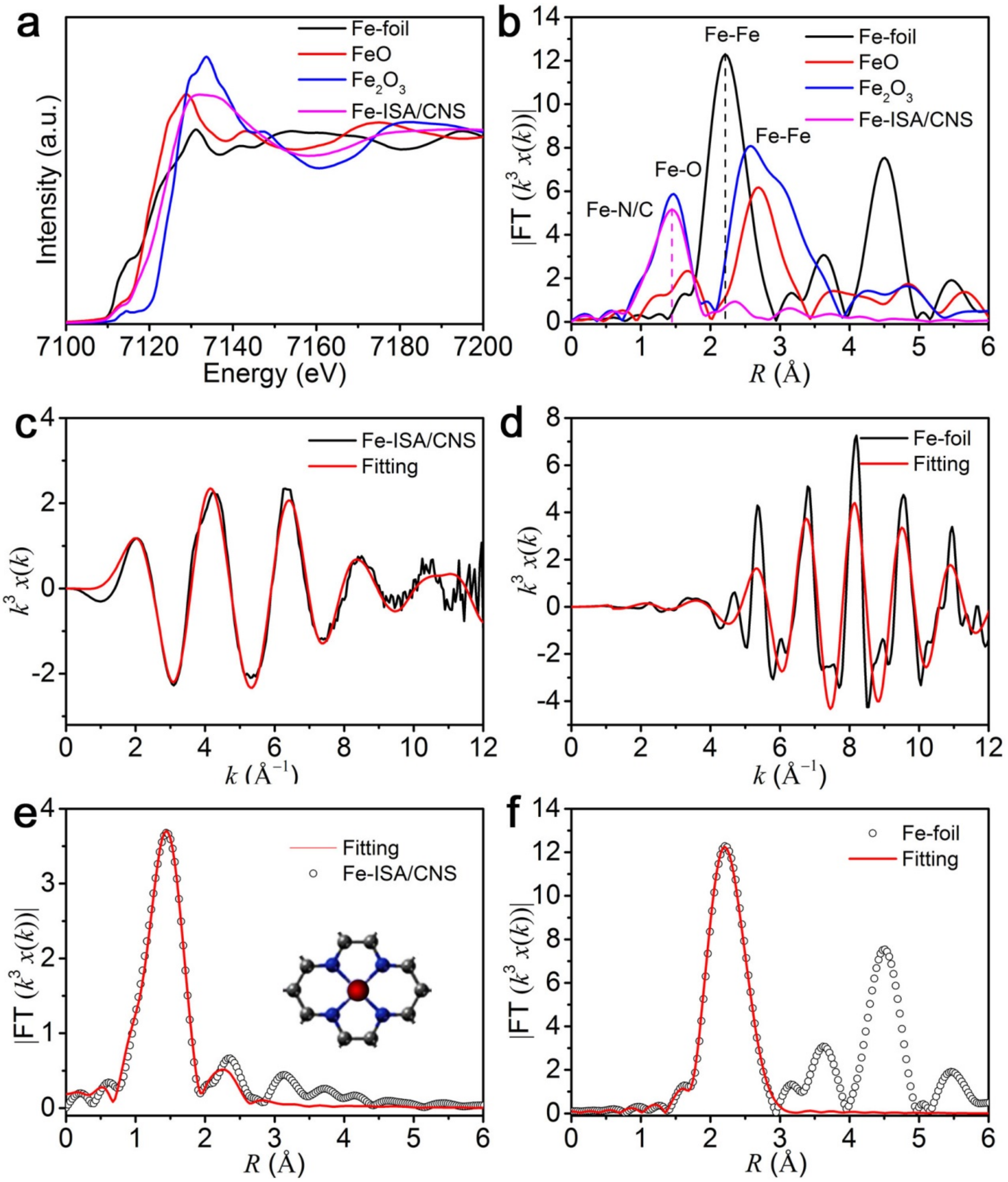

Supplementary Figure 20 | XAFS measurement of Fe-ISA/CNS. (**a**) Fe K-edge XANES spectra. (**b**) Fourier transformed (FT) k^3-weighted χ(k)-function of the EXAFS spectra for Fe K-edge. (**c**) and (**d**) Corresponding EXAFS fitting curves at *k* space. (**e**) and (**f**) Corresponding EXAFS fitting curves *R* space, respectively.

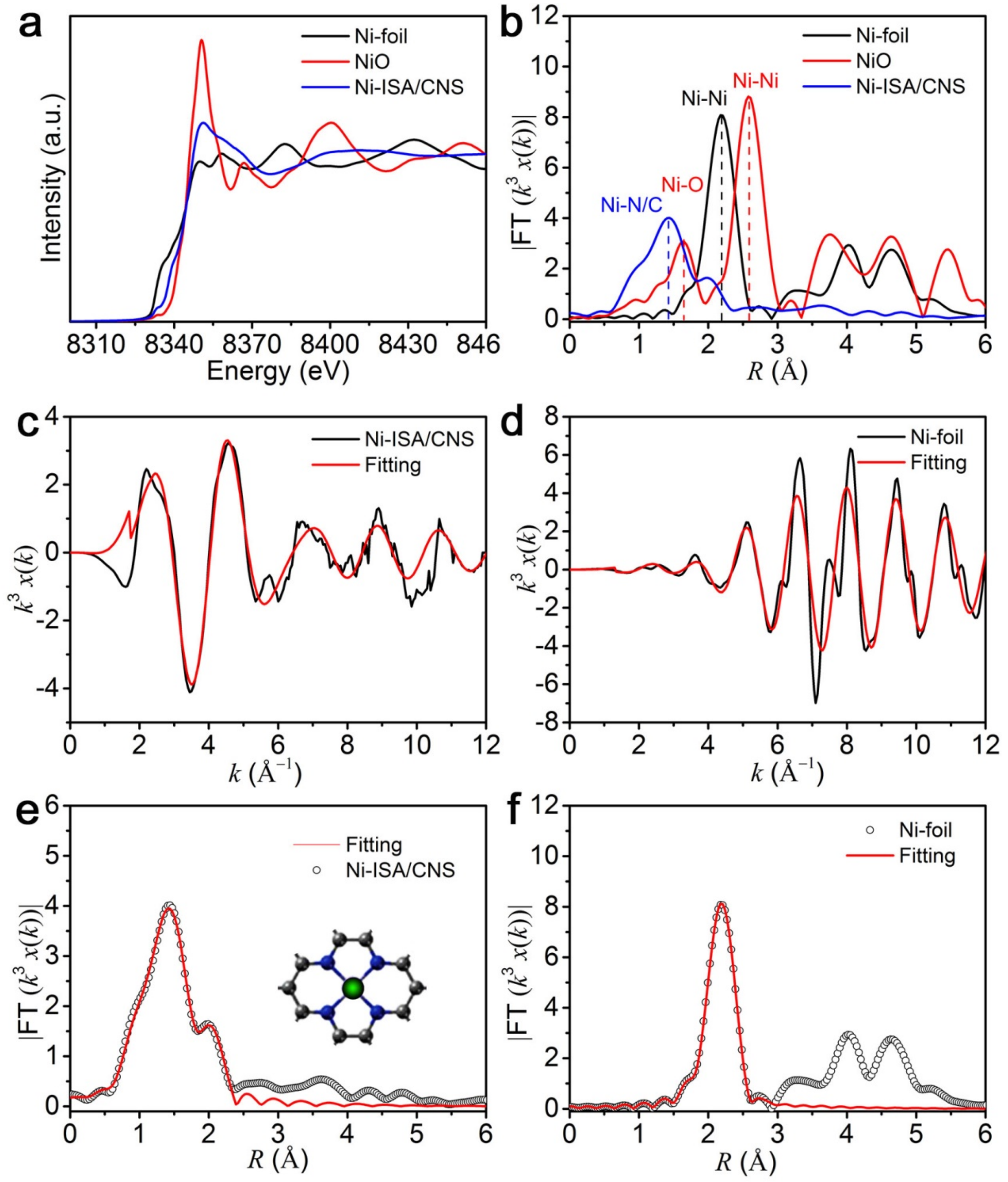

Supplementary Figure 21 | XAFS measurement of Ni-ISA/CNS. (**a**) Ni K-edge XANES spectra. (**b**) Fourier transformed (FT) k^3-weighted $\chi(k)$-function of the EXAFS spectra for Ni K-edge. (**c**) and (**d**) Corresponding EXAFS fitting curves at *k* space. (**e**) and (**f**) Corresponding EXAFS fitting curves *R* space, respectively.

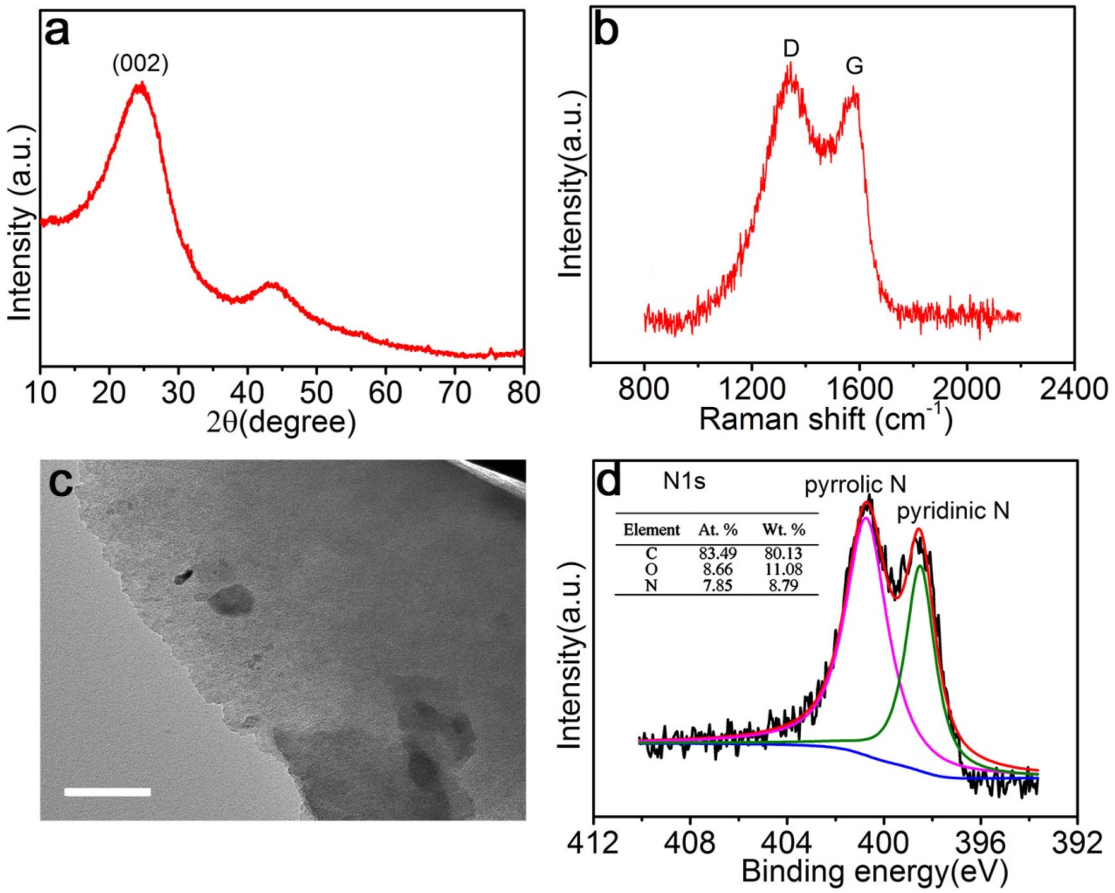

Supplementary Figure 22 | Microstructure and morphology characterizations of N-doped carbon (NC). (**a**) XRD pattern. (**b**) Raman spectrum. (**c**) Typical TEM image. Scale bar, 100 nm. (**d**) XPS N 1s spectra, inset showing the C, O, and N contents.

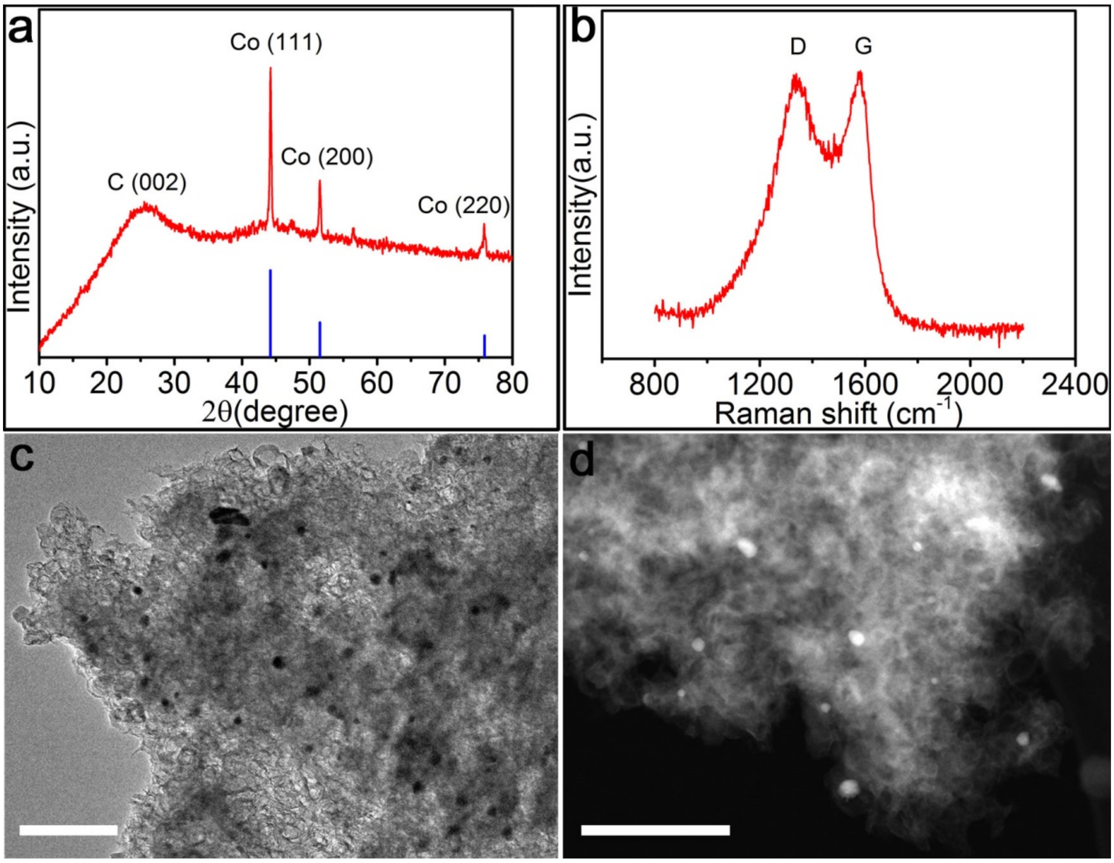

Supplementary Figure 23 | Microstructure and morphology characterizations of Co-NPs. (**a**) XRD pattern with standard Co (JCPDS No. 15-0806, blue vertical line) as reference. (**b**) Raman spectrum. (**c**) Typical TEM image. Scale bar, 200 nm. (**d**) HAADF STEM image. Scale bar, 200 nm.

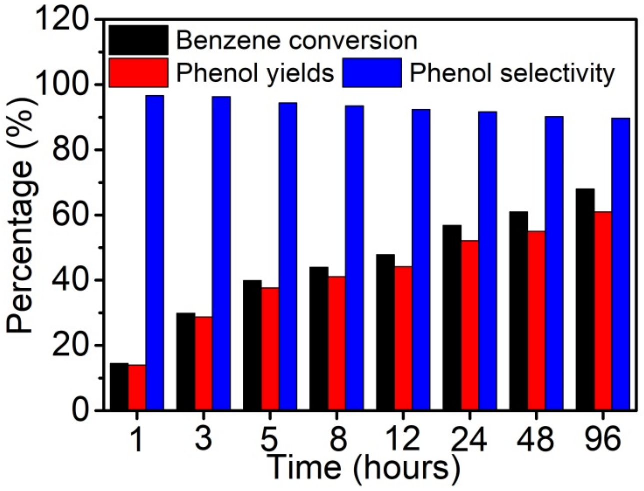

Supplementary Figure 24 | The performance for the direct oxidation of benzene to phenol catalyzed by Co-ISA/CNS catalyst with different reaction times. Reaction conditions: 50 mg catalyst, 0.4 ml benzene, 6 ml H_2O_2 (30%), and 3 ml CH_3CN at room temperature (25 ^oC) for different reaction times.

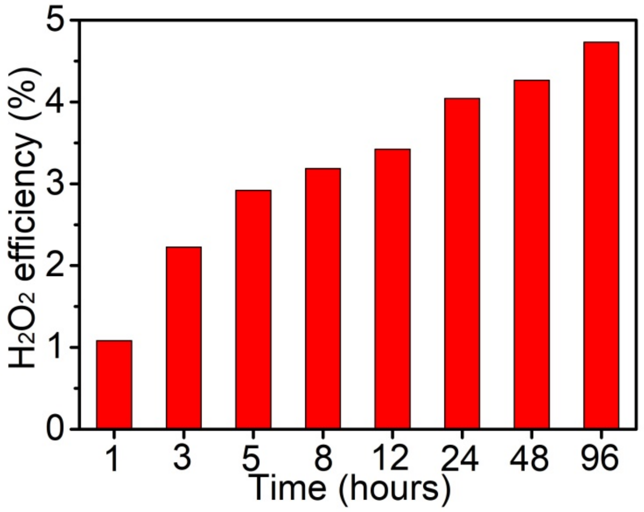

Supplementary Figure 25 | The H_2O_2 efficiency in the direct oxidation of benzene to phenol catalyzed by Co-ISA/CNS catalyst with different reaction times. Reaction conditions: 50 mg catalyst, 0.4 ml benzene, 6 ml H_2O_2 (30%), and 3 ml CH_3CN at room temperature (25 ^oC) for different reaction times. H_2O_2 efficiency = [moles of phenol formed/total moles of H_2O_2 added] × 100.

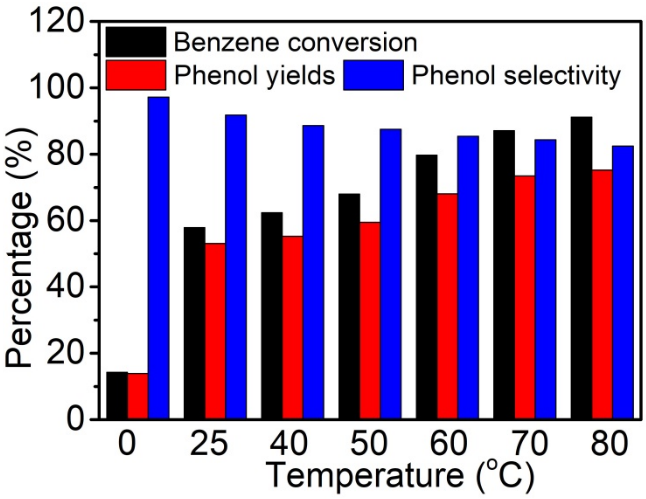

Supplementary Figure 26 | Effect of reaction temperature in benzene oxidation catalyzed by the Co-ISA/CNS catalyst. Reaction conditions: 50 mg catalyst, 0.4 ml benzene, 6 ml H_2O_2 (30%), and 3 ml CH_3CN at different temperature for 24 h.

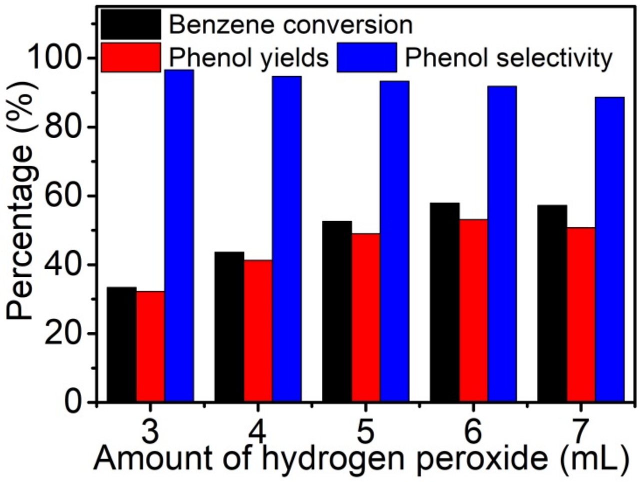

Supplementary Figure 27 | Effect of the amount of H_2O_2 in benzene oxidation catalyzed by the Co-ISA/CNS catalyst. Reaction conditions: 50 mg catalyst, 0.4 ml benzene, different amount of H_2O_2 (30%), and 3 ml CH_3CN at room temperature (25 ^{o}C) for 24 h.

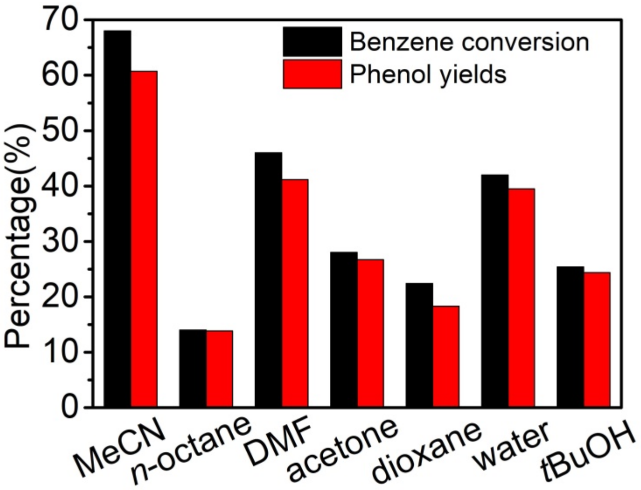

Supplementary Figure 28 | Effect of solvents in benzene oxidation catalyzed by the Co-ISA/CNS catalyst. Reaction conditions: 50 mg catalyst, 0.4 ml benzene, 6 ml H_2O_2 (30%), and 3 ml solvents at room temperature (25 ^oC) for 96 h.

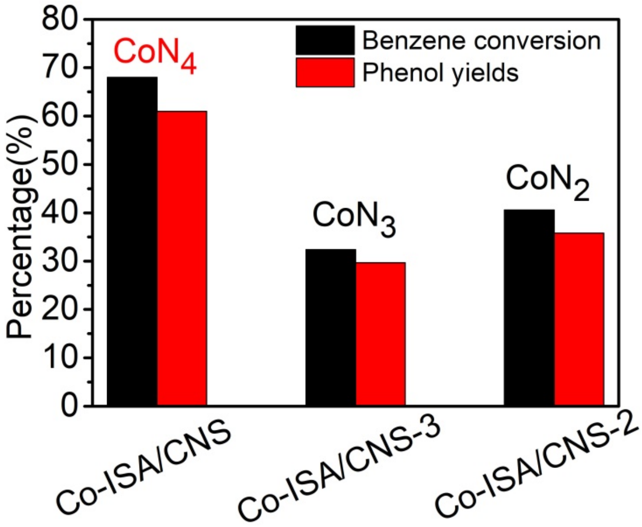

Supplementary Figure 29 | The performance for the direct oxidation of benzene to phenol catalyzed by Co-ISA/CNS catalysts with different N coordination number. Reaction conditions: 50 mg catalyst, 0.4 ml benzene, 6 ml H_2O_2 (30%), and 3 ml CH_3CN at room temperature (25 ^oC) for 96 h.

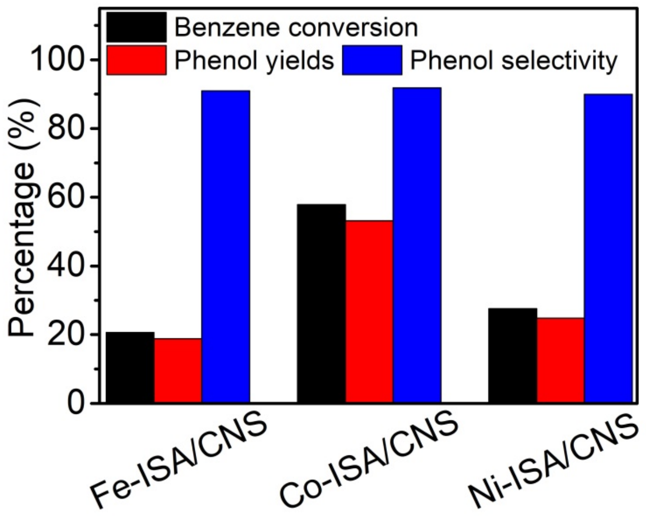

Supplementary Figure 30 | The performance for the direct oxidation of benzene to phenol catalyzed by Fe-ISA/CNS Co-ISA/CNS Ni-ISA/CNS catalysts. Reaction conditions: 50 mg catalyst, 0.4 ml benzene, 6 ml H_2O_2 (30%), and 3 ml CH_3CN at room temperature (25 ^oC) for 24 h.

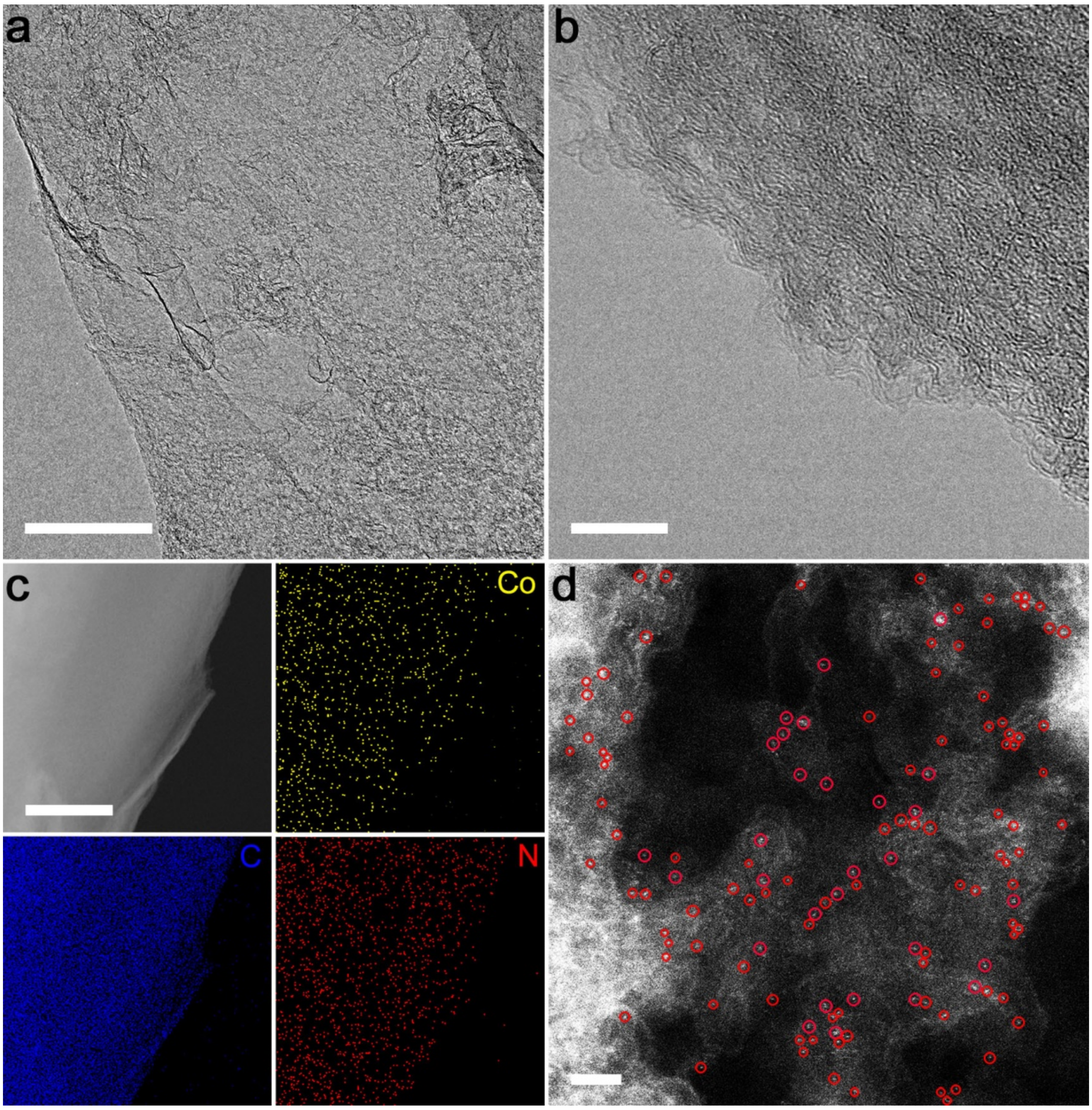

Supplementary Figure 31 | Microstructure and morphology characterizations of recycled Co-ISA/CNS catalyst after recycling reaction. (**a**) Typical TEM image. Scale bar, 200 nm. (**b**) HRTEM image. Scale bar, 10 nm. (**c**) HAADF-STEM image and corresponding EDX elemental mapping. Scale bar, 250 nm. (**d**) Aberration-corrected HAADF-STEM image. Scale bar, 2 nm.

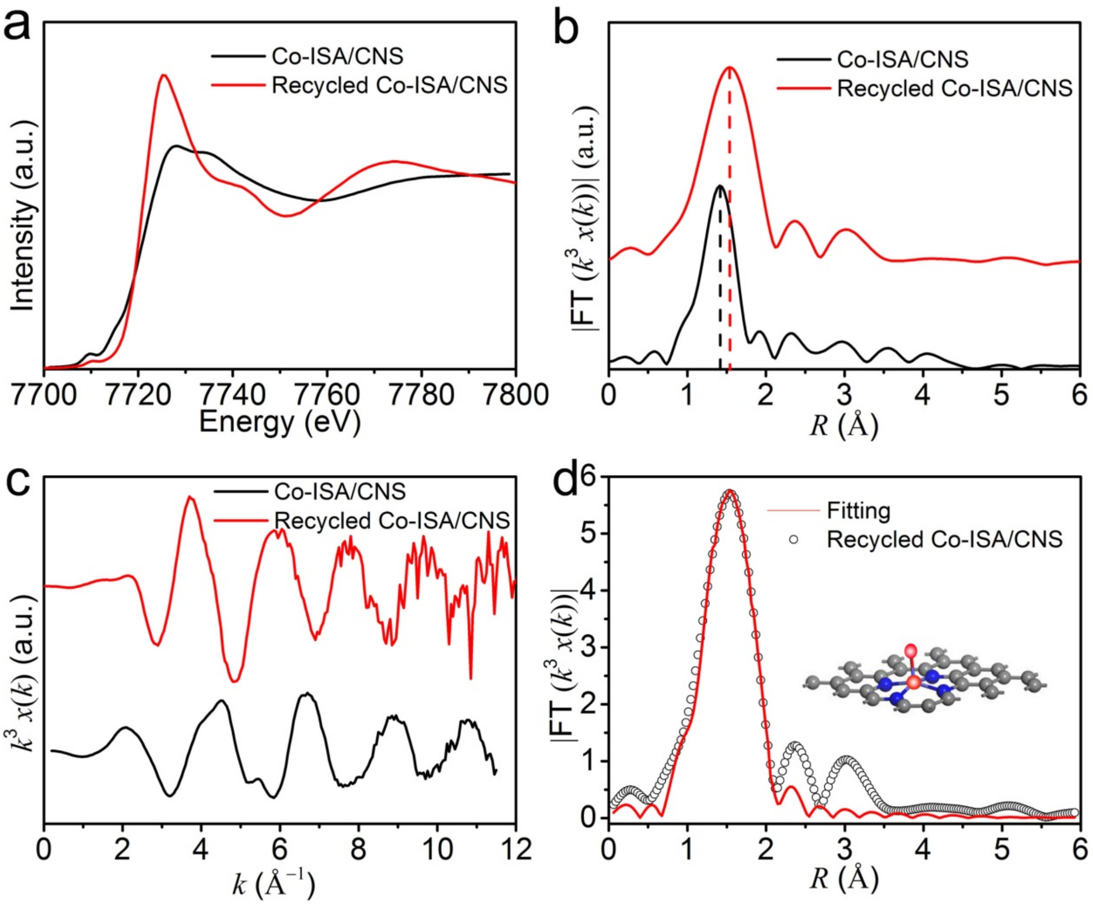

Supplementary Figure 32 | XAFS measurement of recycled Co-ISA/CNS catalyst. (**a**) Co K-edge XANES spectra of the recycled Co-ISA/CNS and fresh Co-ISA/CNS. (**b**) Fourier transformed (FT) k^3-weighted $\chi(k)$-function of the EXAFS spectra for Co K-edge. (**c**) and (**d**) EXAFS curves at *k* space and *R* space, respectively.

The EXAFS spectrum revealed that Co atoms were still atomically dispersed on the N-doped carbon nanosheet after benzene oxidation reaction. Furthermore, the XAFS analysis of recycled Co-ISA/CNS demonstrated the formation of Co=O/O=Co=O intermediates on the CoN_4 center.

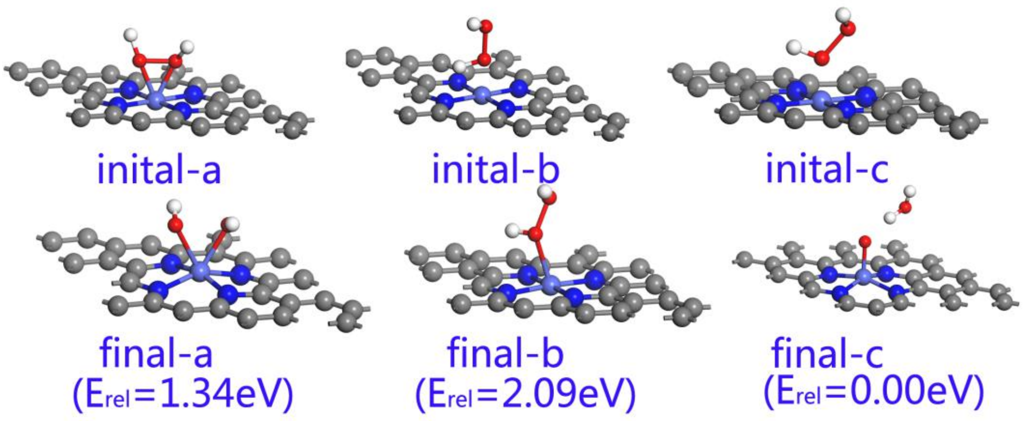

Supplementary Figure 33 | Optimized configurations of H_2O_2 adsorption on Co-ISA/CNS and their corresponding relative adsorption energy. The gray, blue, light blue, red, and white balls represent C, N, Co, O, and H atoms, respectively.

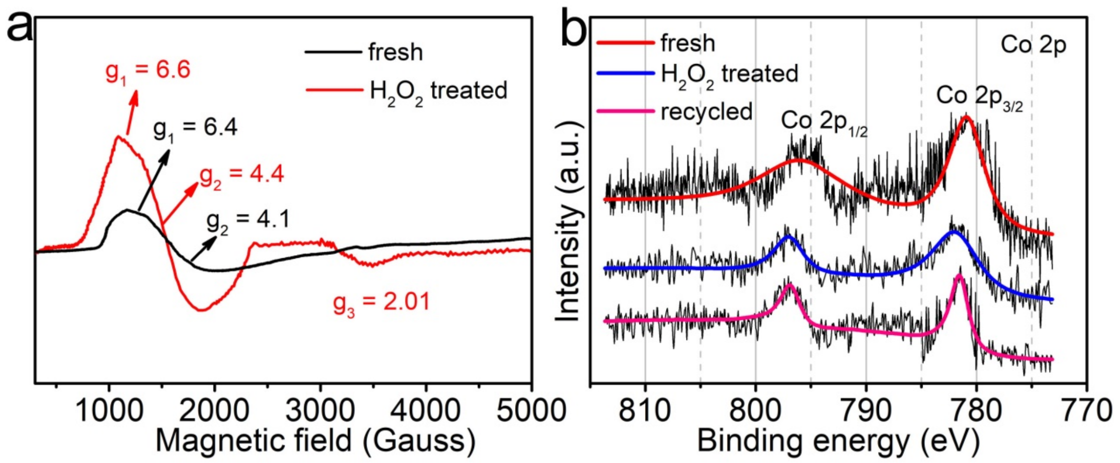

Supplementary Figure 34 | (**a**) EPR spectra of Co-ISA/CNS catalyst with H_2O_2 treatment (red line) in comparison to the corresponding original sample (black line). (**b**) High-resolution Co 2p XPS spectra of Co-ISA/CNS catalyst before and after H_2O_2 treatment.

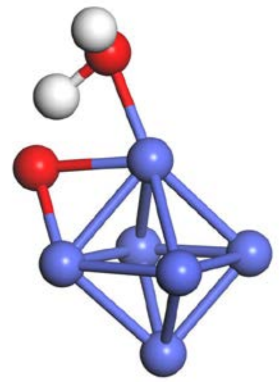

Supplementary Figure 35 | The atomic configuration of the transition state from step 2 to 3 shown in Fig. 4d. The gray, blue, red, and white balls represent C, Co, O, and H atoms, respectively. The interatomic distance of Co-O(H_2O) was calculated to be 1.988 Å, and the interatomic distances between the other O and adjacent Co atoms are 2.526, 1.896, and 2.069 Å, respectively.

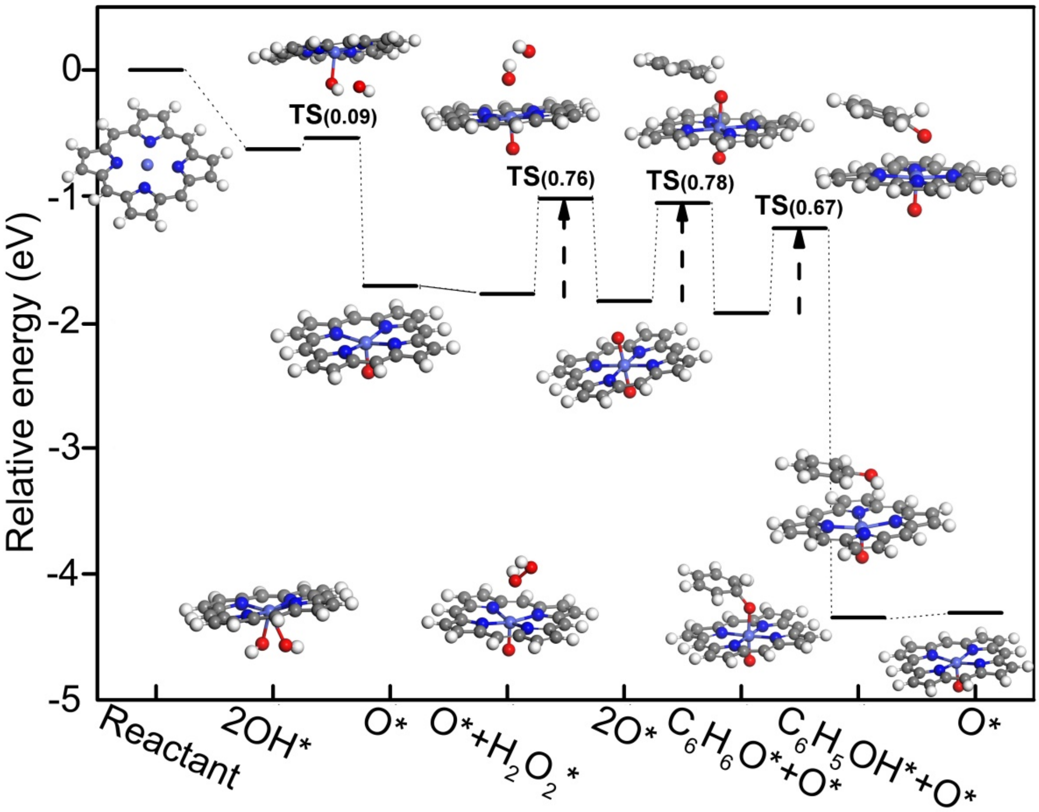

Supplementary Figure 36 | Free energy diagram of the oxidation of benzene to phenol on CoPc. The gray, blue, red, and white balls represent C, Co, O, and H atoms, respectively.

Supplementary Table 1. The elemental compositions of Co-ISA/CNS estimated from XPS and ICP measurements.*

Sample	C (wt %)	O (wt %)	N (wt %)	Co (wt %)
Co-ISA/CNS	80.1	10.1	9.2	0.6

*C, O, and N contents were detected by XPS, and Co contents were determined by ICP.

Supplementary Table 2 Structural parameters of Co-ISA/CNS extracted from the EXAFS fitting. (S_0^2 = 0.90). *

Sample	Scattering pair	CN	R (Å)	$\sigma^2(10^{-3}$ Å2)	ΔE_0(eV)	*R* factor
Co-ISA/CNS	Co-N/C	4.2±0.6	1.98±0.01	9.3±4.2	-2.9±1.8	0.0044
Co-ISA/CNS-3	Co-N/C	3.1±0.4	1.91±0.01	5.3±4.0	-3.2±1.7	0.0041
Co-ISA/CNS-2	Co-N/C	2.2±0.5	1.92±0.01	7.7±4.1	-5.5±1.8	0.0041
Recycled Co-ISA/CNS	Co-N/O	4.8±0.4	2.02±0.01	9.1±4.1	-2.7±1.7	0.0040
Co foil	Co-Co	12[#]	2.49±0.01	6.4±2.2	6.6±0.2	0.0041

* S_0^2 is the amplitude reduction factor; CN is the coordination number; *R* is interatomic distance (the bond length between central atoms and surrounding coordination atoms); σ^2 is Debye-Waller factor (a measure of thermal and static disorder in absorber-scatter distances); ΔE_0 is edge-energy shift (the difference between the zero kinetic energy value of the sample and that of the theoretical model). R factor is used to value the goodness of the fitting. [#]This value was fixed during EXAFS fitting, based on the known structure of Co metal.

Supplementary References

1. Vanderbilt, D. Soft self-consistent pseudopotentials in a generalized eigenvalue formalism. *Phys. Rev. B.* **41**, 7892 (1990).
2. Perdew, J. P., Burke, K. & Ernzerhof, M. Generalized gradient approximation made simple. *Phys. Rev. Lett*. **77**, 3865 (1996).
3. Clark, S. J. *et al.* First principles methods using CASTEP. *Z. Kristallogr*. **220**, 567–570 (2005).
4. Cui, X. *et al.* A graphene composite material with single cobalt active sites: a highly efficient counter electrode for dye-sensitized solar cells. *Angew. Chem. Int. Ed*. **55**, 6708–6712 (2016).
